# Development of a Novel Immune Infiltration-Based Gene Signature to Predict Prognosis and Immunotherapy Response of Patients With Cervical Cancer

**DOI:** 10.3389/fimmu.2021.709493

**Published:** 2021-09-03

**Authors:** Sihui Yu, Xi Li, Jiawen Zhang, Sufang Wu

**Affiliations:** ^1^Department of Obstetrics and Gynecology, Shanghai General Hospital, Shanghai Jiao Tong University, Shanghai, China; ^2^Reproductive Medicine Center, Department of Obstetrics and Gynecology, Shanghai General Hospital, Shanghai Jiao Tong University, Shanghai, China

**Keywords:** cervical cancer, Immunoscore, immune infiltration, bioinformatics, prognostic model

## Abstract

Predictive models could indicate the clinical outcome of patients with carcinoma. Cervical cancer is one of the most frequently diagnosed female malignancies. Herein, we proposed an immune infiltration-related gene signature that predicts prognosis of patients with cervical cancer and depicts the immune landscape as well. We utilized the transcriptome data of The Cancer Genome Atlas (TCGA) and estimated the infiltration level of 28 immune cell types. We screened out four immune cell types conducive to patient survival and recognized their shared differentially expressed genes (DEGs). Four core genes (CHIT1, GTSF1L, PLA2G2D, and GNG8) that composed the ultimate signature were identified *via* univariate and multivariate Cox regression. The optimal model we built up could distinguish patients with cervical cancer into high-score and low-score subgroups. These two subgroups showed disparity in aspects of patient survival, immune infiltration landscape, and response to immune checkpoint inhibitors. Additionally, we found that GTSF1L was decreased gradually along with the severity of cervical lesions, and its potential role in immune contexture and clinical practice were also demonstrated. Our results suggested that the Immunoscore based on four immune-related genes could serve as a supplementary criterion to effectively foresee the survival outcome, tumor infiltration status, and immunotherapy efficacy of cervical cancer patients.

## Introduction

Cervical cancer (CC), as one of the most frequently diagnosed female malignancies, is the fourth leading cause of cancer mortality in females (1). Although current treatment strategies including surgery, chemoradiotherapy, and immunotherapy have tremendously ameliorated the prognosis, the clinical outcome of advanced cervical cancer patients is still not optimistic (2). Owing to its great threat to women’s health and life, exploration of useful prognostic biomarkers and therapeutic targets for cervical cancer seems to be essential.

Mounting evidence indicates that tumor-infiltrating immune cells (TILs) in the tumor microenvironment (TME) participate in tumor progression, aggressiveness, and therapeutic responsiveness (3, 4). A latest single-cell analysis revealed that innate-like CD8^+^ T cells with low cytotoxicity and clonal expansion might account for the compromised antitumor immunity and poor prognosis of liver cancer (5). In cervical cancer, the density of peritumoral CD3^+^ T cells was proven to have the potential for predicting relapse (6), and tumor-infiltrating CD204^+^ M2 macrophages predicted worse prognosis in patients with cervical adenocarcinoma (7). Additionally, increased CD4, CD8, CD20, and CD56 signals were associated with good responders to neoadjuvant chemotherapy (8), and the number of CD8^+^ T cells was correlated with treatment outcome in patients treated with radiotherapy (9). Thus, quantitative molecular signatures closely associated with immune infiltration might display promising capability in predicting the clinical outcome of cervical cancer.

Furthermore, blockade of immune checkpoints such as PD-1/PD-L1 and CTLA-4 has been trendy in malignant tumors (10, 11). Due to the immune components within TME that dampen antitumor immune responses, most tumors often failed to respond in single-agent immunotherapy (12). Therefore, it is necessary to develop superior biomarkers and study the combination therapy for improving the efficacy of immunotherapy.

Within this context, we established a superior predictive model incorporating multiple biomarkers in cervical cancer and put forward a novel modeling algorithm to construct our new immune infiltration-based gene signature. This signature distinguishes patients with cervical cancer in respect of clinical outcome, tumor infiltration state, and immunotherapy efficacy, which may help to improve patient management and enable personalized treatment.

## Materials And Methods

### Retrieval of Transcriptome Data and Immune Cell Infiltration Analysis

Transcriptome profiling data harmonized to fragments per-kilobase million (FPKM) of cervical cancer from TCGA (https://tcga-data.nci.nih.gov/tcga/) for the cervical squamous cell carcinoma and endocervical adenocarcinoma (CESC) project were downloaded. We also obtained gene transfer format (GTF) files from Ensembl (http://asia.ensembl.org) for annotation of the mRNAs. Moreover, 217 early cervical cancer tissues (IB1, GSE44001) based on the GPL14951 (Illumina HumanHT-12 WG-DASL V4.0 R2 expression beadchip) platform and corresponding information of disease-free survival (DFS) were both retrieved from the GEO database (http://www.ncbi.nlm.nih.gov/geo). Besides, the gene expression profile of 128 cervical tissue specimens (GSE63514) based on the GPL570 (Affymetrix Human Genome U133 Plus 2.0 Array) platform was also downloaded. Subsequently, a list of representative marker genes of tumor-infiltrating immune cell types was acquired from Charoentong’s research involving 366 microarrays of immune cells summarized from 37 studies, which was used for immune cell infiltration analysis (13).

### Acquisition of Clinical Information of Patients

Basic clinical information of patients with CESC was retrieved from the CESC project of TCGA. We excluded samples whose overall survival (OS) time or survival status was not available. After filtering, a total of 304 patients with CESC from the TCGA dataset were enrolled in this study.

### Construction of an Immune-Related Signature to Evaluate the Immunoscore

We developed a novel computational frame to identify tumor-infiltrating immune-related signature by integrating immune and transcriptome profiling analysis of CESC tumors as follows.

To identify the DEGs, we used *R* package *limma* for differential expression analysis among these genes. The thresholds were settled as log fold change (FC) >1 along with adjusted *p*-value < 0.05. For the four immune cell types, DEGs in common were obtained as candidate immune-related genes. To enhance the availability of this model, we constructed a 1-or-2 matrix to represent the expression levels of these overlapped genes in patient samples. Assuming Y as the value of gene A, Y is defined as 1 if the expression level of gene A in the individual sample is lower than its median expression level in all cases; otherwise, Y is assigned as 2.

Then, we screened out the core genes by a Cox regression strategy. A univariate analysis was conducted, following which a multivariate Cox proportional hazard regression was performed. Candidate genes with *p*-value < 0.05 were selected for establishment of the model. The 1-, 3-, and 5-year ROC curves were plotted. The following formula was applied to calculate the Immunoscore for all clinical samples: Tumor-infiltrating Immunoscore$=\sum_{i=1}^{N}Exp_{i}\cdot Gene_{i}$, where N is the total number of core immune-related genes, Exp*_i_* is the regression coefficient, and Gene*_i_* refers to the relative expression of gene *i* transformed by our expression matrix. According to the median value, the whole cohorts were re-divided into high- and low-score subgroups.

### Validation of the Established Immunoscore Signature

Kaplan–Meier analysis was used to illustrate the survival difference of patients from high-score and low-score subgroups. In line with our expectation, higher Immunoscore indicated a better prognosis for patients, as was visualized by *R* tools. The *R* packages employed in this step involved *survival, survminer*, and *plyr*. In addition, *ggrisk* package was also applied to re-order these clinical samples in TCGA datasets as well as GSE44001. The relationship between four hub genes consisting this immune signature and prognosis was demonstrated with risk curve, scatter plot, and heatmap.

We also visualized this signature model with a nomogram, and further analysis revealed that this signature could serve as an independent prognostic predictor, which was proved by univariate and multivariate Cox regression analyses. The R packages involved in this operation included *survival, survMisc, pROC, survminer*, and *rms*.

### Correlation Analysis Between Immune Landscape and the Constructed Signature

Spearman correlation analysis was performed between the Immunoscore and infiltrating immune cells. The correlation coefficients of the results were shown in the lollipop diagram, with a significance threshold of *p*-value < 0.05. The *R* package *ggplot2* was used in this procedure.

The violin and box plot was used for visualization and was labeled as listed below: < 0.0001 = ****, < 0.001 = ***, < 0.01 = **, and < 0.05 = *. Wilcoxon signed-rank test was applied to evaluate the statistical differences among different subgroups of Immunoscore.

### Investigation of Immune Subtypes

To analyze the clinical cases from another perspective, we recognized immune subtypes based on their immune infiltration status among 28 immune cells, adopting the currently acknowledged method. The *R* package *ConsensusClusterPlus* was applied to identify the subtypes and *sigclust* proved that the *p*-value was significant in this classification. We chose *k* = 5 and plotted the survival map of five immune subtypes *via R* packages *survminer* and *survival.*

### Estimation of the ICI-Related Immune Molecule Expression

To study the immune infiltration characteristics, we amounted the expression levels of ICP and ICD genes in high- and low-score groups of the established model, respectively. The differences in these gene expressions were analyzed by Wilcoxon signed-rank test and displayed in a violin chart. The *gglot2* and *ggpubr* packages were performed to visualize this plot.

### The Predictive Potential of Immunoscore on Clinical Response

To verify the clinical application potential of this signature, we conducted the Kruskal–Wallis test to explore the relationship between the Immunoscore and clinical outcome. The box diagram was used for visualization of the result. The *R* packages *ggplot2* and *ggpubr* were utilized.

We also explored the significance of the Immunoscore in clinical immunotherapy treatment. The prognostic difference among four subgroups was compared by Kruskal–Wallis test and the results were shown as survival curves *via survival* and *survminer* of *R*.

### Gene Set Enrichment Analysis

To evaluate the infiltration of immune cells, we performed single-sample gene set enrichment analysis (ssGSEA) using the marker gene set of different immune cells with *R* package *GSVA*, which can calculate the normalized enrichment score of immune cell types. Furthermore, GSEA was also conducted with Bioconductor packages *clusterProfiler* and *msigdbr*, which can identify hallmark gene sets or immunologic signatures that are activated or suppressed according to their correlation with Immunoscore and immune-related genes. Moreover, we evaluated the correlation between this Immunoscore and specific phenotypes such as epithelial–mesenchymal transition (EMT) and hypoxia *via* the ssGSEA method. Related gene sets were downloaded from the Molecular Signature Database (MSigDB) *via* Gene Set Enrichment Analysis tool (GSEA, http://software.broadinstitute.org/gsea/index.jsp).

### TIMER2.0 Platform Analysis

The correlation of the mutation status of specific genes (MUC16, ERBB2, KRAS, and MAPK1) with GTSF1L expression was analyzed by TIMER2.0 (14). The association between immune infiltrates and genomic changes in TCGA-CESC project was also explored using the TIMER2.0 sCNA module.

### Kaplan–Meier Plotter Online Analysis

To analyze the prognostic value of the four hub genes, the online analysis tool Kaplan–Meier plotter (http://kmplot.com/analysis/) was utilized to shed light on the correlation of their expression levels with patients’ relapse-free survival (RFS) based on the TCGA cohort (15).

### Cell Culture

Human cervical epithelial cell line H8 and human cervical cancer cell lines HeLa, SiHa, ME180, and Caski were purchased from FuHeng Cell Center (Shanghai, China). These cells were cultured in DMEM (HyClone, UT, USA) supplemented with 10% fetal bovine serum (FBS, Gibco, USA) in 100 U/ml penicillin/streptomycin (Beyotime, Jiangsu, China). All cell lines were cultured at 37°C with 5% CO_2_. Cell cultures were periodically screened for mycoplasma contamination.

### RNA Isolation, cDNA Synthesis, and Real-Time PCR

We applied the TRIzol reagent (Invitrogen, USA) to extract RNA from cell lines and each total RNA was then reversely transcribed into cDNA using PrimeScript RT-PCR kit (Takara, China) and subsequently amplified by TB Green™ Premix Ex Taq™ II (Takara, China) according to the manufacturer’s protocol. The expressions of mRNAs were further normalized to GAPDH. The sequences of primers used in this study are listed in **Table S1**. The relative expressions were quantified by the 2^−ΔΔCt^ method.

### Statistical Analysis

All data were processed within the R software (version 4.0.4). The Kruskal–Wallis test was applied to compare among at least three groups, whereas the Wilcoxon test was used to compare between two groups. The Kaplan–Meier plotter was conducted to generate survival curves for subgroups of the cohort, and the log-rank test was utilized to estimate the statistically significant differences. Spearman analysis was employed to calculate the correlation coefficient. For all analyses, a two-tailed *p* < 0.05 was regarded as statistically significant.

## Results

### The Landscape of Immune Cells in Human Cervical Cancers

**Figure 1:**
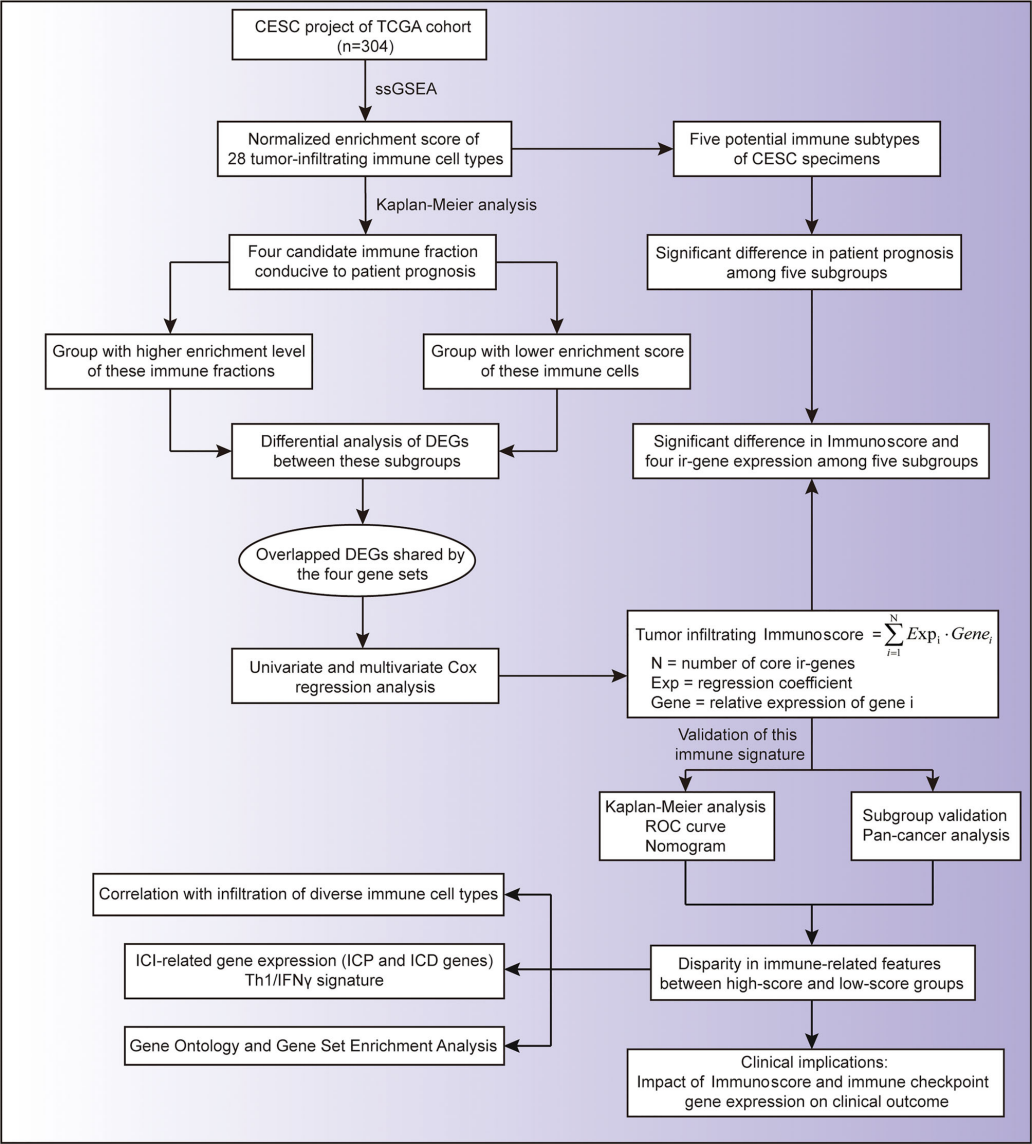
Flow chart of this study.

To explore the effect of different immune cell subgroups on cervical cancer patient’s prognosis, we first obtained the transcriptome profiling data of the CESC project of the TCGA database, of which 304 tumor samples were included. The entire flow diagram of this study is shown in **Figure 1**.

**Figure 2:**
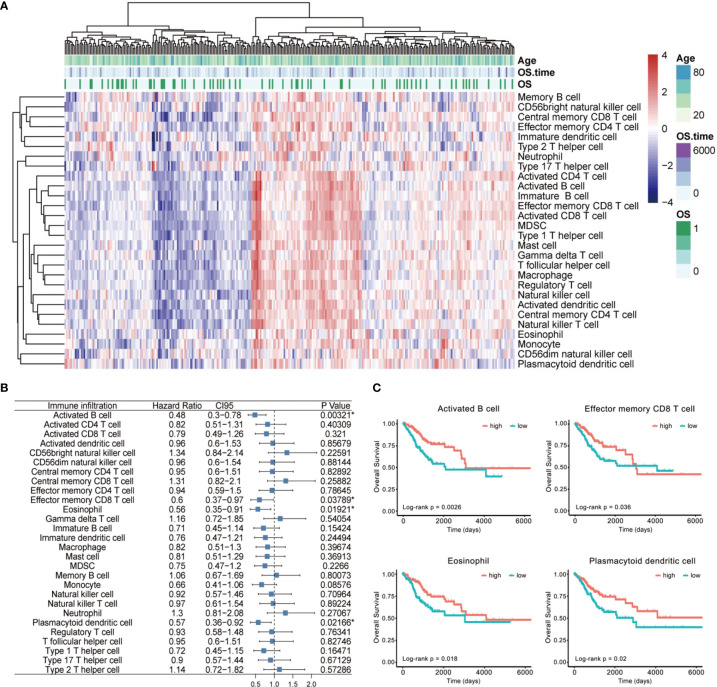
The landscape of immune cell infiltration of CESC. **(A)** The heatmap for normalized enrichment scores of 28 immune cell types in cervical cancer of the TCGA cohort. Rows represent tumor-infiltrating cells, and columns represent samples. **(B)** A forest map showed four immune cells of prognostic value identified by Cox proportional hazard regression in the stepwise method. **(C)** Kaplan–Meier curves for overall survival (OS) of all CESC patients with the four identified immune cell-infiltrating classes. Log-rank test showed *p* < 0.05 respectively. *p < 0.05.

Then, we annotated these data with GTF files of Ensembl, calculated the normalized enrichment score of various immune cells by single-sample GSEA analysis to characterize their expression pattern in CESC cases (**Figure 2A**), and explored their impact on patient prognosis (**Figure 2B**). We totally identified four immune cells positively correlated with OS (**Figures 2B, C**), including activated B cell (*p* = 0.00321, HR = 0.48, 95% CI [0.3–0.78]), effector memory CD8 T cell (*p* = 0.03789, HR = 0.6, 95% CI [0.37–0.97]), eosinophil (*p* = 0.01921, HR = 0.56, 95% CI [0.35–0.91]), and plasmacytoid dendritic cell (*p* = 0.02166, HR = 0.57, 95% CI [0.36–0.92]).

### Establishment of an Immune-Related Cox Regression Model of Prognostic Value

**Figure 3:**
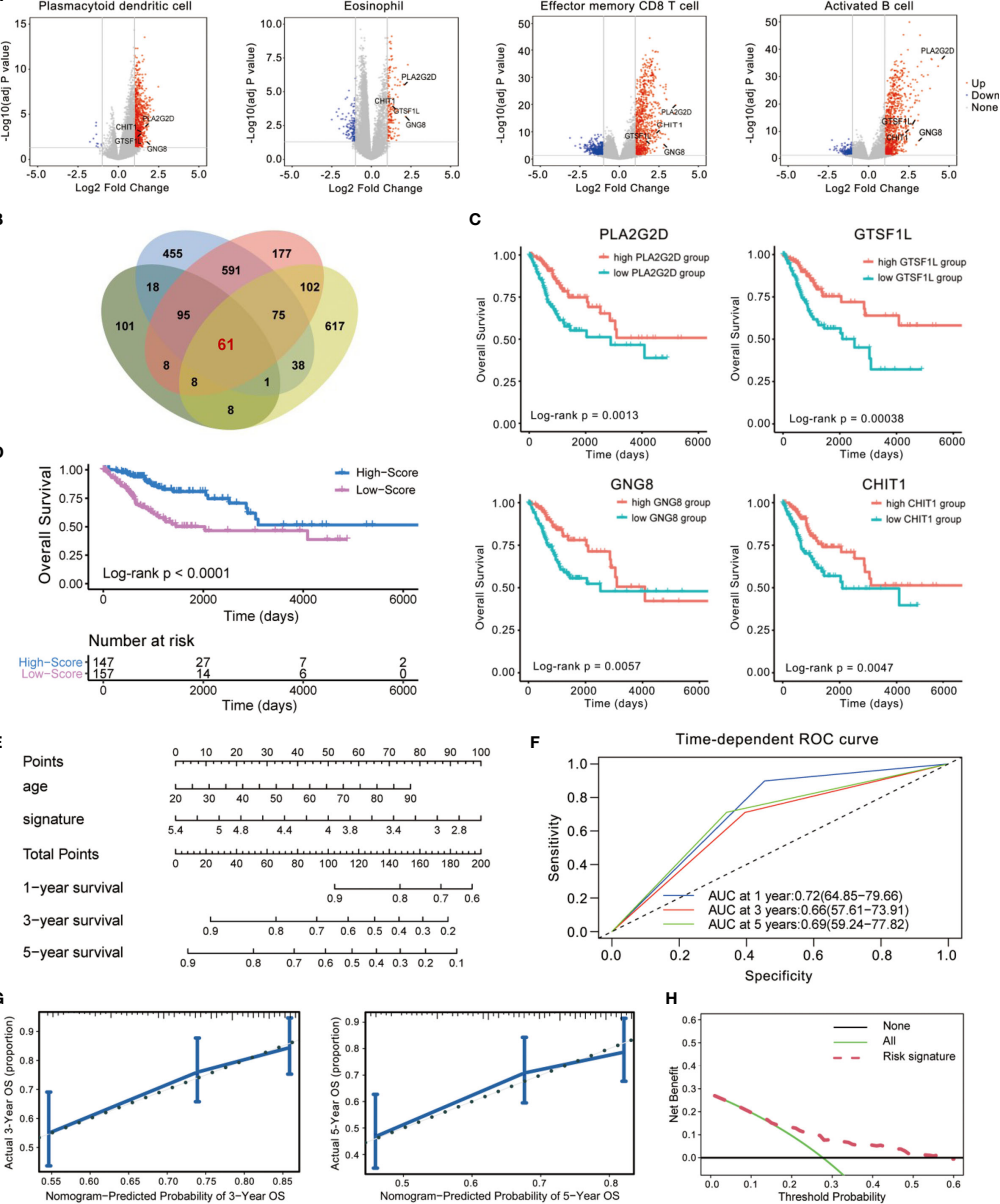
Establishment of an immune-related prognostic signature using Cox regression analysis. **(A, B)** Identification of differentially expressed genes (DEGs) using TCGA cohorts and annotation of Ensembl. The volcano plot **(A)** and Venn diagram **(B)** are shown. **(C)** Kaplan–Meier curves for overall survival (OS) of all CESC patients with the four immune-related genes. Log-rank test showed *p* < 0.01, respectively. **(D)** Patients in the high-score group displayed a longer overall survival time tested by Kaplan–Meier test. **(E)** Construction of a nomogram combining Immunoscore with clinical feature (age) for survival prediction. The Immunoscore presented as an independent prognostic predictor by multivariate Cox regression. **(F)** The 1-, 3-, and 5-year ROC of the optimal model suggested that the AUC values were approximately 0.70. **(G)** Calibration plot showing that nomogram-predicted survival probabilities corresponded closely to the observed proportions. **(H)** The decision curve analysis (DCA) of the immune signature.

To screen for the immune-related genes of these four immune cell types mentioned above, we distinguished those genes showing significantly disparate expression levels between the high- and low-infiltration subgroup (**Figure 3A**) and identified 61 differentially expressed genes (DEGs) in common (**Figure 3B**). After re-examining their tendency of upregulation or downregulation in the subgroups of the four immune cells, we eventually confirmed 60 genes that were highly expressed in the high-infiltration subgroup. These genes were assumed to be universally relevant to immune cells and critical for maintenance of immune cellular functions, and thus have been considered as candidate CESC model components (**Table S2**).

Then, we used a 1-or-2 matrix to represent the relative expression levels of the overlapped 60 genes and investigated the association between the expressions of these genes and patients’ OS time in the TCGA dataset *via* univariate Cox proportional hazards regression analysis. As listed in **Table S2**, we validated 23 candidates that impose effect on patient survival. A multivariate Cox regression analysis was then implemented following this single-factor test, which extracted a set of four immune-related genes (CHIT1, GTSF1L, PLA2G2D, and GNG8) and constituted a Cox proportional hazards model by stepwise method (**Table S2**).

These four genes were all upregulated in the high-infiltration group of the four immune cells (**Figure 3A**), positively related to one another (**Figure S1**), and significantly correlated with CESC patients’ OS (**Figure 3C**). Therefore, we consisted these four genes as a potential prognostic signature. The signature was indicative of immune infiltration using the expression of the four immune-related genes weighted by the multivariate Cox regression coefficient as follows: Immunoscore = (0.7071341 × expression value of CHIT1) + (0.6469606 × expression value of GNG8) + (0.5702634 × expression value of GTSF1L) + (0.7482603 × expression value of PLA2G2D). Taking advantage of this model, we calculated the Immunoscore to all these acceptable cases of patients with CESC from TCGA.

The median Immunoscore was identified as the cutoff point to re-classify the cohort into two subgroups: 147 samples were classified into the high-score group and 157 were classified into the low-score group. Kaplan–Meier analysis demonstrated that patients in the high-score group were inclined to exhibit a longer survival time than those in the low-score group (Log-rank *p* < 0.0001, **Figure 3D**). We also demonstrated this immune-related signature with an easy-to-use and clinically adaptable risk nomogram. As depicted in **Figure 3E**, higher total points based on the sum of assigned numbers for each factor in this nomogram was associated with a worse 1-, 3-, and 5-year OS rates. The prognostic performance of this signature after adjusted by another clinical factor (age) showed that Immunoscore was still significantly correlated with favorable OS in the cohort (*p* < 0.0001, HR = 0.53, 95% CI [0.41–0.70], **Table S3**). Moreover, we calculated the areas under curve (AUCs) for the 1-, 3-, and 5-year receiver operating characteristic (ROC) curve of this model. As shown in **Figure 3F**, the AUC values of this signature were 0.72, 0.66, and 0.69 at 1, 3, and 5 years of OS, indicating a relatively high reliability.

We also computed the discrimination index as well as the calibration plot of the model for 3- and 5-year survival (**Figure 3G**). The accompanied C-statistic discriminatory index value of 0.708 reveals that this signature is quite robust in distinguishing subjects with different outcomes. Moreover, the calibration plots of this nomogram showed excellent concordance between observed outcome and predicted survival probabilities. The decision curve analysis (DCA) also indicated that the risk signature brought clinical benefit to patients with CESC (**Figure 3H**). These results further foster the clinical implication of the prognostic signature.

### Validation of the Four-Gene Immune-Related Prognostic Model

**Figure 4:**
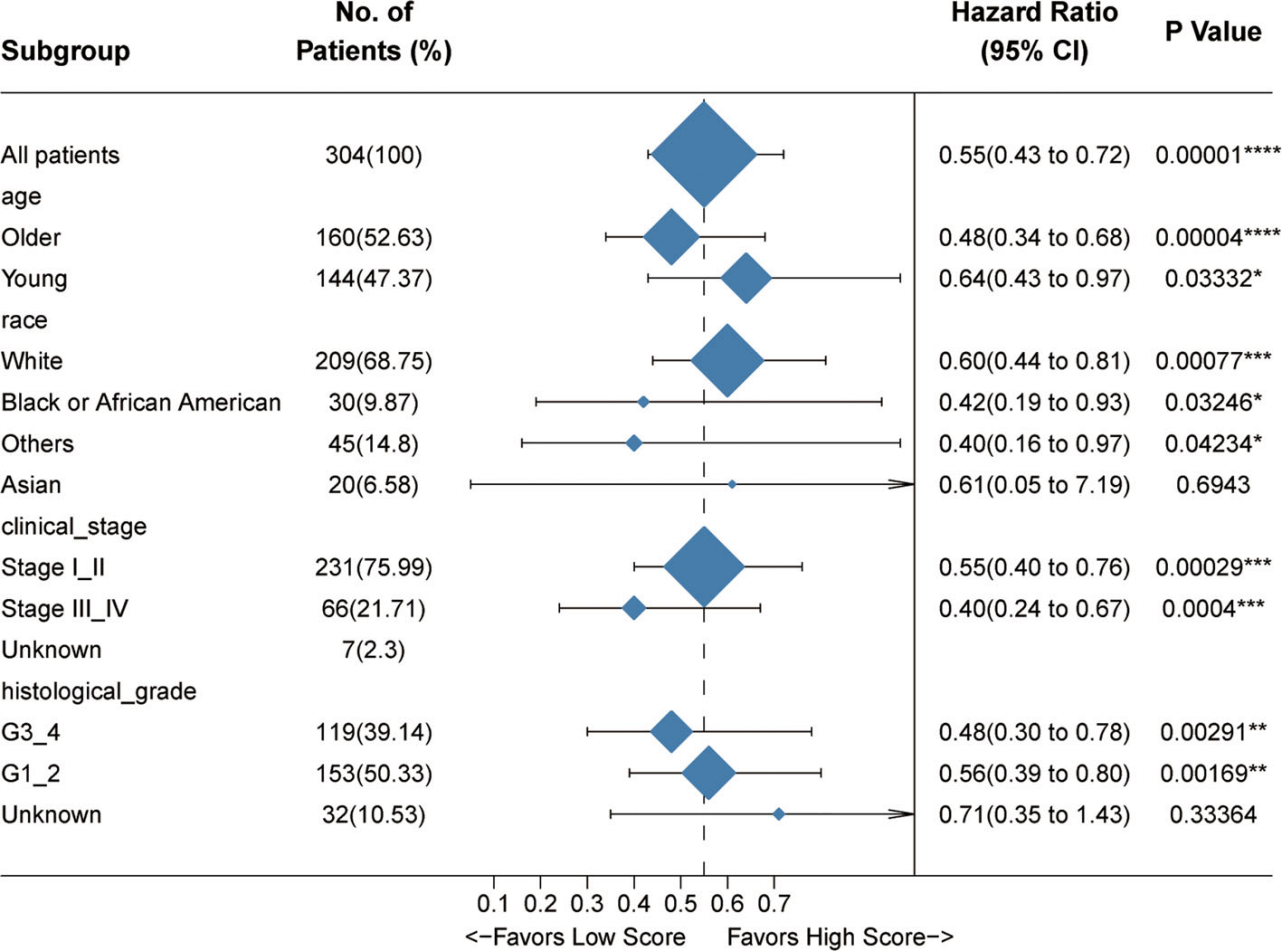
Internal validation of the prognostic potential of the Immunoscore. A univariate Cox hazard ratio analysis revealed that the Immunoscore was statistically different in almost all the subgroups classified by age, race, clinical stage, and histological grade. *p < 0.05, **p < 0.01, ***p < 0.001, ****p < 0.0001.

To confirm the robustness of this signature, we tested its prognostic power by using subgroup analysis as well as pan-cancer evaluation. According to the clinical characteristics involving age, clinical stage, histological grade, and human race, the whole cohort was divided into different subgroups. The association of the Immunoscore with OS in these subgroups was examined *via* univariate Cox analysis. As expected, the four-gene Immunoscore predicts a superior clinical outcome (**Figure 4**).

**Figure 5:**
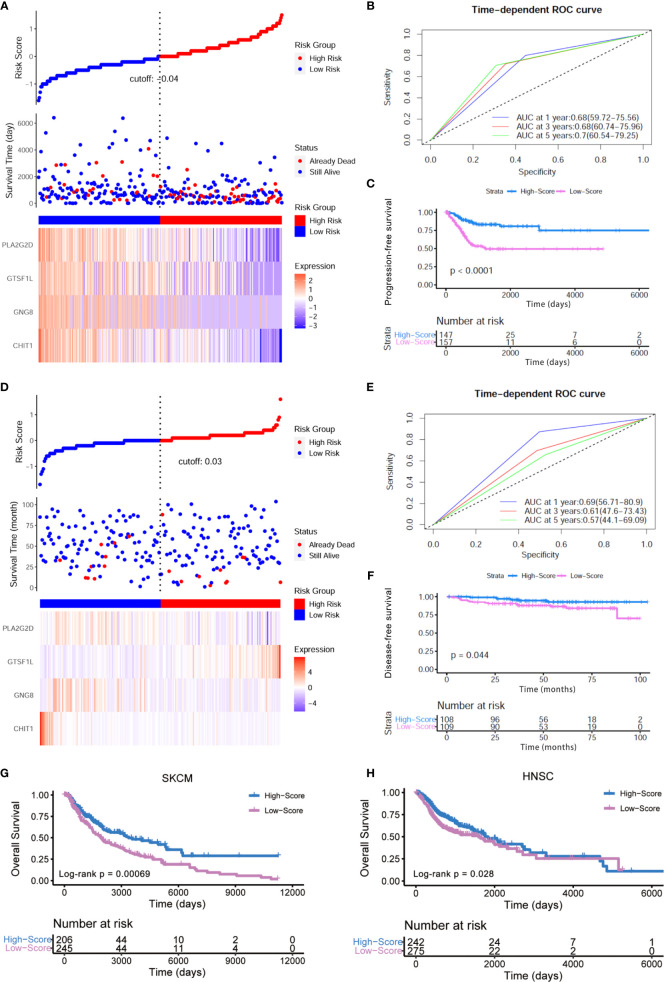
External verification of prognostic value of this Immunoscore. **(A)** The risk curve and scatter plot of each sample in the TCGA-CESC cohort after realignment *via ggrisk* algorithm. The heatmap showed distinct expression profiles of four hub genes in the high- and low-risk groups. **(B)** The 1-, 3-, and 5-year ROC of the optimal model based on the progression-free survival of TCGA-CESC. **(C)** Patients in the high-score group displayed a longer progression-free survival time tested by Kaplan–Meier analysis. **(D)** The risk curve and scatter plot of each sample in the GSE44001 cohort (IB1 stage, *n* = 217) after realignment *via ggrisk* algorithm. The heatmap showed distinct expression profiles of four hub genes in the high- and low-risk groups. **(E)** The 1-, 3-, and 5-year ROC of the optimal model based on the disease-free survival (DFS) of GSE44001 (IB1 stage, *n* = 217). **(F)** Patients in the high-score group displayed a longer disease-free survival time tested by Kaplan–Meier analysis. **(G, H)** Kaplan–Meier curves for high- and low-score groups in the TCGA-SKCM (Log-rank test, *p* < 0.001) and TCGA-HNSC cohorts (Log-rank test, *p* < 0.05).

Meanwhile, we also explored the impact of this Immunoscore on the progression-free survival (PFS) and RFS of patients from the TCGA-CESC cohort. In light of the risk curve obtained by *ggrisk* R package, we could find that the four hub genes showed a relatively higher expression in the low-risk group with better outcome (**Figure 5A**). ROC curve (**Figure 5B**) and Kaplan–Meier analysis (Log-rank *p* < 0.0001, **Figure 5C**) also verified the predictive power of this Immunoscore. Higher expressions of these four immune-related genes indicated ameliorated PFS and RFS, respectively (**Figures S2A, B**). There also existed statistically significant differences between high- and low-score groups in terms of the enrichment score of gene sets correlated with hypoxia and EMT (**Figures S3A, B**). To further underpin our conclusion, we testified this established Immunoscore in an additional series of patients with early cervical cancers (IB1 stage, *n* = 217). Consistent results were achieved that this Immunoscore predicted a superior disease-free survival time in this cohort (**Figures 5D** **–F**). Experimental validation was conducted to quantify the relative expressions of these four hub genes in diverse human cervical cell lines. The results revealed that all of these four genes exhibited lower expression levels in cervical cancer cells compared to human cervical epithelial cell H8 (**Figure S4**).

In addition, we conducted a pan-cancer analysis on the basis of TCGA transcriptome profiling data and clinical information. Among the 33 sorts of cancers in the TCGA cohort, we extended our conclusion to examine whether this model posed an impact on other human cancers. The pan-cancer analysis revealed significant association between the four-gene Immunoscore and OS in two other cancers: head and neck squamous cell carcinoma (HNSC, *p* = 0.00847, HR = 0.81, 95% CI [0.7–0.95]) and skin cutaneous melanoma (SKCM, *p* = 0.00005, HR = 0.73, 95% CI [0.63–0.85]) (**Figure S5A**). In HNSC and SKCM, the signature could stratify patients into low score and high score with significantly different OS using the same Immunoscore derived from the training CESC dataset. Furthermore, Kaplan–Meier analysis also showed that a high score of this model predicted a better prognosis in HNSC (*p* = 0.028) and SKCM (*p* < 0.001) patients (**Figures 5G, H**). Additionally, these four hub genes all displayed higher expression levels in the low-risk group with longer OS, which was re-divided by *ggrisk* package (**Figures S5B, C**). The univariate Cox hazard ratio analyses in sub-cohorts of SKCM and HNSC revealed that the Immunoscore was statistically different in almost all the subgroups classified by age, gender, and tumor stage in SKCM (**Figure S6A**) and in patients within subgroups such as younger age, male patients as well as III–IV clinical stage in HNSC (**Figure S6B**). These results indicated that our Immunoscore model exhibited some specificity to squamous cancers to a certain extent.

**Figure 6:**
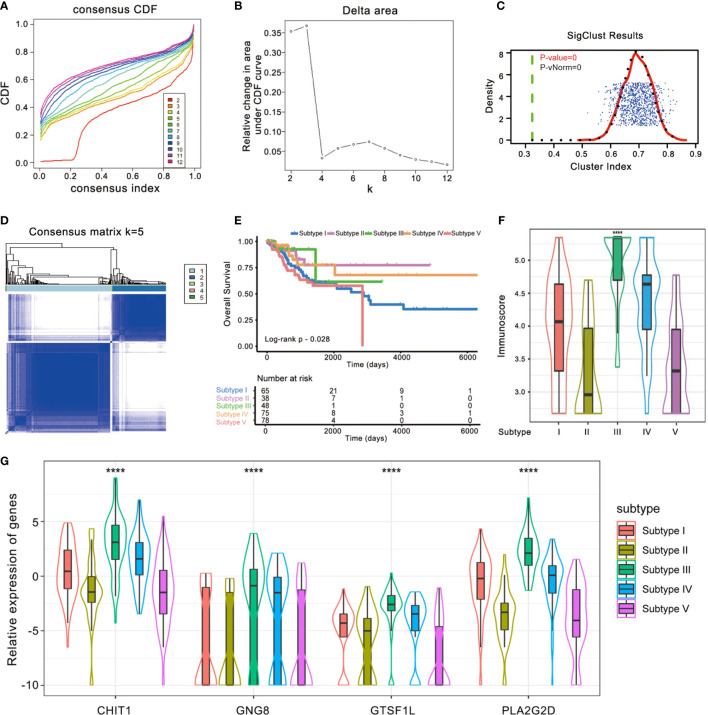
Identification of immunogenic subtypes of the CESC cohort. **(A–D)** Unsupervised clustering of CESC samples based on their tumor-infiltrating patterns to classify patients into five subgroups. **(E)** Kaplan–Meier curves for the five groups of patients. The Log-rank test showed an overall *p* = 0.028. **(F)** The Immunoscore of five immune subtype clusters. The statistical difference of five clusters was compared through the Kruskal–Wallis test. ****p < 0.0001. **(G)** Difference in the four immune-related gene expressions among distinct immune clusters (Kruskal–Wallis test, *p* < 0.0001).

Meanwhile, we re-classified the TCGA cohort of 304 patients based on the enrichment score of 28 immune subpopulations *via R* package *ConsensusClusterPlus* (**Figures 6A** **–C**). The whole cohort was classified into five subtypes (**Figure 6D**), and Kaplan–Meier analysis demonstrated that these groups were significantly different in OS (Log-rank *p* = 0.028, **Figure 6E**): subtype V showed a worse outcome whereas subtypes III and IV implied a better prognosis. We also found that there existed a significant difference in Immunoscore among the five immune subtypes (Kruskal–Wallis test *p* < 0.0001, **Figure 6F**), suggesting that the immune-related signature is closely associated with the immune microenvironment of CESC. In addition, the expression levels of the four immune-related genes in this signature seemed to be significantly different among five subtypes as well: they tended to be overexpressed in immune subtypes III and IV, while they had relatively lower expression levels in immune subtype V (**Figure 6G**).

### Characterization of the Immune Landscape Disparity With Immunoscore Signature

**Figure 7:**
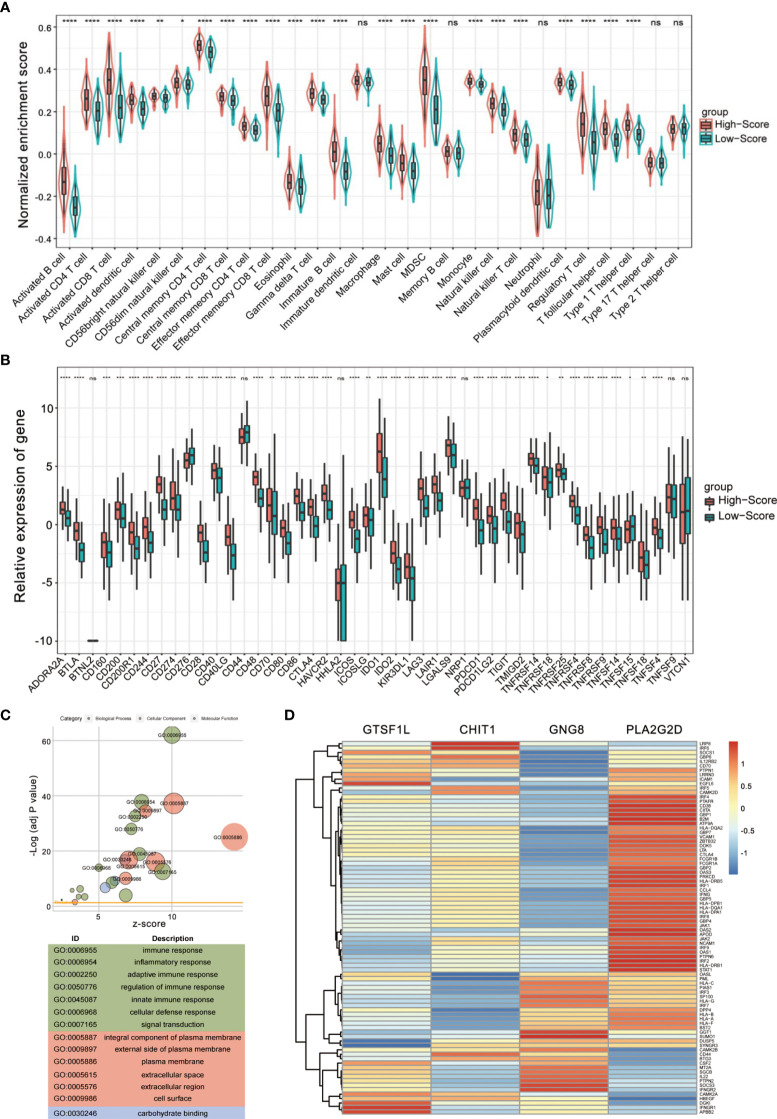
Evaluation of tumor-infiltrating landscape and immune checkpoint inhibitor-related molecules by the Immunoscore. **(A)** Patients in the high-score group were more positively associated with tumor-infiltrating immune cells including CD4^+^ T cells, CD8^+^ T cells, and T follicular helper cells. The statistical difference of two groups was compared through the Wilcoxon test. *p < 0.05; **p < 0.01; ***p < 0.001; ****p < 0.0001, ns, not significant. **(B)** High Immunoscore was positively associated with increased immune checkpoint levels such as PDCD1, CTLA4, and LAG3 in patients with CESC. The statistical difference of two groups was compared through the Wilcoxon test. *p < 0.05; **p < 0.01; ***p < 0.001; ****p < 0.0001, ns, not significant. **(C)** Gene Ontology (GO) enrichment analysis of the differentially expressed genes between high- and low-score groups. **(D)** A heatmap showing the correlation between the four immune-related genes and Th1/IFNγ signature.

Since the signature was composed of immune-related genes, the association between the Immunoscore and intratumoral immune cell infiltration was further explored. We re-divided the cases into high-score and low-score subgroups and estimated the discrepancy in tumor-infiltrating immune cells with distinct groups. The infiltration of 28 immune subpopulations of high-score and low-score groups was estimated using single-sample GSEA analysis. As shown in **Figure 7A**, patients in the high-score group were more inclined to be enriched with the vast majority of immune subpopulations, such as CD4^+^ T cells, CD8^+^ T cells, and T follicular helper cells, while patients with a low score showed obviously less enrichment. Spearman correlation analysis also indicated that a higher score of this signature corresponded to greater immune cell infiltration (**Figure S7**).

Immune checkpoint inhibitors (ICIs) have been administered for CESC treatment in clinical practice (16), and we found that a series of immune checkpoints (ICPs) had a positive impact on CESC prognosis (**Figure S8**). In view of the importance of ICPs and immunogenic cell death (ICD) modulators in tumor immunology, we compared the expression level of these genes in different groups (**Figures 7B** and **S9**). According to the plot, most ICI-related biomarkers, ICP and ICD genes, showed significantly relatively high expression levels in the high-score group, including PDCD1, LAG3, IDO1, CTLA4, and CD274 (*p* < 0.0001, **Figure 7B**).

To further confirm whether the Immunoscore was highly reflective of the immune infiltration status, we then performed Gene Ontology (GO) enrichment analysis. The bubble plot suggested that differential expression between high- and low-score groups were significantly enriched in diverse immune-related biological processes (**Figure 7C**). Furthermore, GSEA conducted with *R* package *clusterProfiler* and *msigdbr* also identified hallmark gene sets or immunologic signatures that are activated or suppressed according to their correlation with Immunoscore and the four immune-related genes (**Figures S10**, **S11**).

Since Th1 bias is principally responsible for the activation of antitumor immune response and the Th1/IFNγ gene signature is associated with clinical outcome (17), we examined the relationship between the four immune-related genes and a combined Th1/IFNγ gene signature. As shown in **Figure 7D**, the four immune-related genes exhibit positive correlation with the Th1/IFNγ signature. These findings suggest that the signature composed of the four immune-related genes seemed to be extensively positively correlated with almost all immune cell types and might be essential for basic immune cellular functions.

### Potential of the Signature as an Indicator of Immunotherapy Response in Patients With CESC

**Figure 8:**
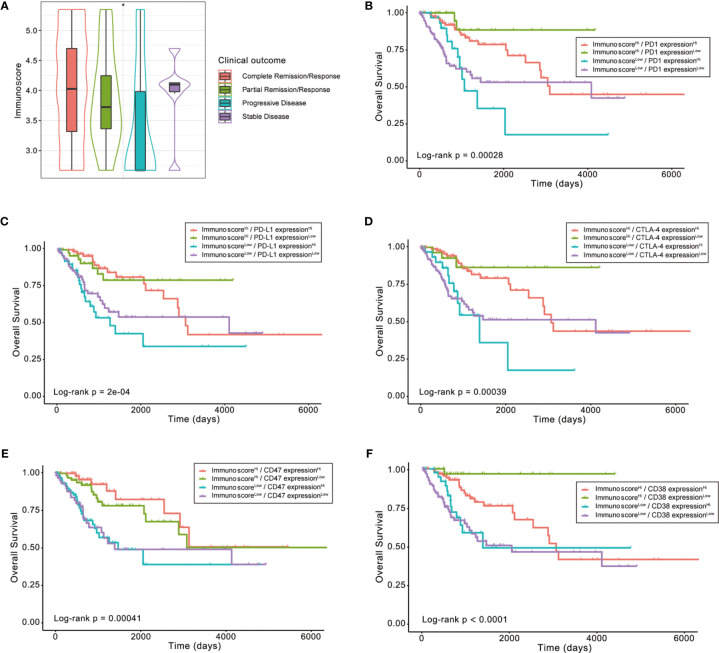
The role of Immunoscore in the prediction of immunotherapeutic benefits. **(A)** Difference in Immunoscore distribution between patients with different outcomes after first course of treatment (Kruskal–Wallis test *p* < 0.05). **(B–F)** Kaplan–Meier curves for patients in the TCGA-CESC cohort stratified by both Immunoscore and expressions of immune checkpoints, such as PD1, PD-L1, CTLA-4, CD47, and CD38. Log-rank test, *p* < 0.001, *p < 0.05.

Next, we retrieved the clinical information of TCGA-CESC samples and found significant differences in Immunoscore distribution between patients with different outcomes after the first course of treatment (Kruskal–Wallis test *p* < 0.05, **Figure 8A**). Notably, patients with progressive disease had the lowest Immunoscore, whereas patients showing stable disease or complete response had a substantially higher Immunoscore.

Then, we explored whether the immune infiltration posed an impact on clinical outcomes in patients with similar expression levels of ICI-related genes. The patients were stratified by the Immunoscore as well as the high or low ICI-related gene expression into four groups, among which their survival distribution was compared. As shown in **Figure 8B**, patients with high Immunoscore and high PD-1 displayed prolonged OS compared to those with low Immunoscore and high PD-1 (Log-rank *p* < 0.001). Similar survival patterns could also be observed using the Immunoscore and PD-L1, CTLA-4, CD47, or CD38 (**Figures 8C** **–F**).

### The Role of GTSF1L in Immune Contexture and Clinical Practice

**Figure 9:**
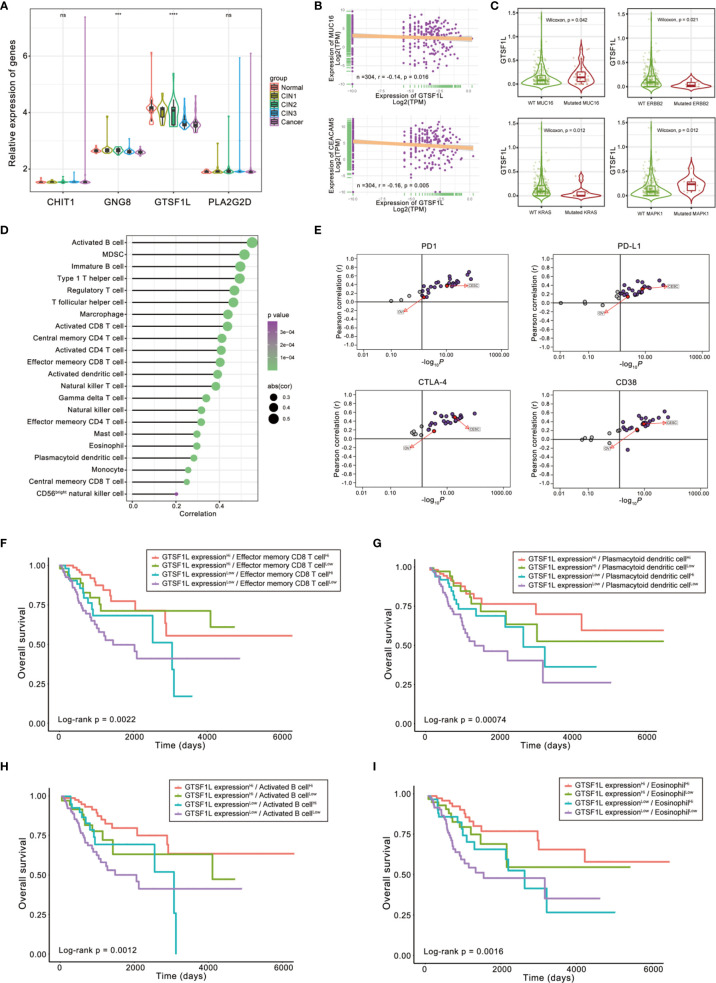
The role of GTSF1L in immune contexture and clinical practice. **(A)** Differences in the expressions of the four immune-related genes in GSE63514, which involves 128 cervical specimens, ranging from normal cervix to cervical cancer (Kruskal–Wallis test, ***p < 0.001; ****p < 0.0001, ns, not significant). **(B)** Scatterplots depicting the negative correlation between GTSF1L expression and clinical biomarkers involving CA125 (MUC16) and CEA (CEACAM5) in the TCGA-CESC cohort. The Spearman correlation is shown (*p* < 0.05). **(C)** Difference in GTSF1L expression among specific gene-mutated conditions such as MUC16, ERBB2, KRAS, and MAPK1 (Wilcoxon test, *p* < 0.05). **(D)** Spearman correlation analysis of GTSF1L expression and tumor-infiltrating cells. **(E)** The Pearson correlation analysis of GTSF1L expression and immune checkpoints, such as PD1, PD-L1, CTLA-4, and CD38, in all sorts of cancers of TCGA. **(F–I)** Kaplan–Meier curves for patients in the TCGA-CESC cohort stratified by both GTSF1L expression and infiltration levels of the four prognostic immune cells. Log-rank test, *p* < 0.01.

To further analyze the expression patterns of the four immune-related genes in different pathological types of cervical tissues, we compared the expressions of these four genes in an independent GEO dataset GSE63514, which involves 128 cervical specimens, ranging from normal cervix to cervical cancer. The results demonstrated that GTSF1L and GNG8 exhibited a gradually decreasing tendency along with the disease progression, and GTSF1L showed greater expression disparity (**Figure 9A**).

CA125 (MUC16), CEA (CEACAM5), and HE4 (WFDC2) are clinically established diagnostic and prognostic markers of CESC; high levels of these biomarkers suggest cancer progression and poor prognosis (18, 19). Here, we found that the expression level of GTSF1L in CESC displayed a significantly negative relationship with these biomarkers (**Figures 9B** and **S12A**), which was relatively consistent with the better prognosis observed in the GTSF1L-high patient group.

Besides, the expression level of GTSF1L had a significant difference in some specific gene-mutated conditions. For instance, GTSF1L was upregulated in CESC with mutated CA125 or MAPK1 compared to the wild-type counterpart, while the expression of GTSF1L was reduced in the KRAS- or ERBB2-mutated CESC tumors (**Figure 9C**). According to the online tool TIMER 2.0, we also found that high amplication of GTSF1L was linked to higher infiltration of CD8^+^ naive T cell and resting mast cell, but correlated with lower infiltration of NK cell and activated mast cell (**Figure S13**). In addition, GTSF1L as well as other candidate immune-related genes (CHIT1, GNG8, and PLA2G2D) were all universally positively correlated with infiltration of the majority of immune cells (**Figures 9D** and **S14**).

The correlation between GTSF1L and ICI-related genes such as PD1, PD-L1, CTLA4, and CD38 was computed by Pearson analysis in all types of cancers from the TCGA cohort. The results revealed that GTSF1L was on the whole positively correlated with these biomarkers in multiple cancers, especially in CESC and OV (**Figures 9E** and **S12B**).

Finally, we stratified patients with high GTSF1L expression based on immune cell infiltration level and found that immune cell infiltration indicated noticeable survival differences in patients with high GTSF1L expression (**Figures 9F** **–I**). Meanwhile, survival difference was also present in patients with low GTSF1L expression when stratifying them with signature-based immune cell infiltration level. Furthermore, we plotted the survival distributions of patients stratified by GTSF1L expression as well as ICI-related gene expression and obtained a similar tendency to that of the immune signature (**Figure S15**). These observations suggested that GTSF1L might be a predictive biomarker of treatment response to immunotherapy.

## Discussion

In this study, we attempted to establish a predictive model with four immune-related genes with an expression matrix of 1-or-2 to replace their previous specific expression values. To a certain extent, we avoided the problem caused by platform differences and detecting technique to a certain extent, so as to make up for the limitations of previous studies (20–25).

First, we retrieved the transcriptome data of CESC samples from the TCGA cohort, analyzed the normalized enrichment score of diverse immune cells. Then we performed a differential expression analysis to identify DEGs and validated core genes using a univariate analysis combined with multivariate Cox hazard regression method, on the basis of a 1-or-2 expression matrix. In this way, we determined the formula of Immunoscore signature and calculated the AUC value of 1-, 3-, and 5-year ROC curves. With the median score set as the cutoff point, we differentiated the high- and low-score group among patients with CESC. Furthermore, we evaluated this novel signature in the context of survival, tumor-infiltrating immune cells, and checkpoint-related biomarkers.

Our algorithm suggested a significant immune-related gene model in which researchers were only required to compare the expression level of samples with median expression of each gene so as to avoid the disparity of detecting platform and technique. This signature has clinical significance after distinguished into high- and low-score groups. Since these genes were all closely related to immune cells, they are likely to participate in the process of immune context regulation as well as immune activation. The results revealed that the two groups possessed distinct survival outcome, tumor immune infiltration, and ICI-related biomarker expression. We also validated this modeling algorithm in subgroups of the CESC dataset of the TCGA project, according to different clinicopathological characteristics. Besides, this conclusion could also be examined in SKCM and HNSC datasets of the TCGA cohort, which implied that this modeling algorithm worked well. Therefore, these proposed core genes might be novel biomarkers for further study.

To further understand the prognostic role of this signature, we utilized this Immunoscore to analyze patient survival, leading to significant risk stratification of OS in patients with CESC. With single-sample GSEA analysis to measure the infiltration levels of 28 immune cells of different groups of Immunoscore, we also uncovered significantly different immune context between two groups. The high-score group defined by this signature seems to be relatively immune-inflamed with greater immune cell infiltration whereas the low-score group seems to be an immune-cold group with less infiltration of immune cells. Thereby, the results indicated that this signature is highly reflective of immune cell infiltration giving rise to a better prognostic outcome in patients with CESC.

Cancer cells often increase the expression of molecules involved in the inhibitory immune checkpoints to escape anti-tumor immunity (26, 27). Nowadays, immunotherapy targeting PD-1 or other immune checkpoints elicits antitumor responses in cervical cancer (16, 28). Nevertheless, clinical responses to checkpoint blockade immunotherapy vary due to disparities in elements such as tumor mutational burden (TMB) or cytolytic elements of TME; therefore, not all patients are suitable for ICI treatment based on their diverse immune context *in vivo* (29, 30). A pre-existing intratumor adaptive immune response was required for effective immunotherapy, such as checkpoint inhibitors. For instance, specific immune contexture with higher immune gene expression as well as Immunoscore (“hot-inflamed” tumors) was associated with reduced risk of certain diseases such as colorectal cancer (31). Therefore, an early assessment and prediction for ICI response by biomarkers is in urgent need for selection of patients likely to benefit from ICIs (32–34).

Here, we found that patients with high levels of our Immunoscore were inclined to express higher ICI-related genes (PD1, PD-L1, CTLA-4, CD38, and CD47). This tendency does not coincide with the currently universal opinions that correlate higher expression of ICI genes to poor clinical outcomes. Actually, although the high-score group co-expressed higher levels of immune-resistant molecules such as PD1, PD-L1, and CTLA-4, due to its tight correlation with immune infiltration, the immune cell subpopulations were still more activated. On the other hand, the low-score group exhibited a more exhausted immune landscape compared to the high-score group, despite that the expression levels of immune-resistant markers were relatively lower in this subgroup. A study pinpointed that in spite of the traditionally recognized role as exhaustion T-cell markers, PD-1, LAG-3, and TIM-3 were expressed preferentially in activated TILs (35). This result was consistent with a model where co-inhibitory receptors were upregulated upon T-cell stimulation so as to limit exaggerated responses and tissue damage. However, additional studies are still warranted to refine the phenotype of cells expressing each inhibitory receptor combination and explore how to apply this fact into clinical intervention.

Immune infiltration in TME is regarded as a crucial factor of immunotherapy response (3, 36). Combined estimation of biomarkers independently predicting response has been better-studied in recent decades. A refined set of biomarker tools stratifying patients with the same sort of cancer into different immune-based patterns of tumor immunobiology may allow more rational and personalized treatment regimens. In this study, we noticed that the Immunoscore has a discriminatory power in patients with similar expression levels of immune checkpoint genes. This phenomenon also revealed the complicated crosstalk between immune infiltration and immune checkpoint genes in TME and patient prognosis. In this perspective, the immune signature is also implied to be associated with better response to ICI therapy.

On the other hand, we are aware of pre-existing studies, which have developed some immune-related prognostic models to demonstrate the relationship between immune landscape and cervical cancer development. For instance, Zhao et al. (25) and Ding et al. (24) investigated the differentially expressed immune-related genes in CESC. However, the association of their model with immune infiltration was not definitely pinpointed and had nothing to do with clinicopathological characteristics. In four latest published studies, researchers have reclassified the cervical cancer cohorts and established gene signature derived from the ESTIMATE algorithm or constructed specific TMEscore based on the penetration pattern of immune cells to evaluate the relationship between immune infiltration and prognosis (20–23). Considering the relatively great differences between tumor and normal tissues, we aimed at exploring the heterogeneity in immune landscape of tumors. Notably, we expected to find the crucial immune-related genes of prognostic value, which might not be necessarily within the list of known immune-related gene sets as previous studies employed (24). Therefore, we analyzed the prognostic value of 28 immune cells and identified the principal common DEGs of these prognostic immune cells *via* multivariate Cox analysis to develop a new Immunoscore signature. We thoroughly illustrated the characteristics of this signature from multiple angles, such as immune cell infiltration, ICI-related gene expression, Th1/IFNγ signaling, and GO and GSEA analysis. In addition, we also evaluated the impact of this Immunoscore and ICI-related gene expression on different clinical outcomes.

Our study also had some limitations. For example, the detailed supporting information of a few patients in the TCGA cohort was relatively lacking, which hindered a more comprehensive analysis of clinicopathological characteristics for patients with CESC. Moreover, due to the relative scarcity of cervical cancer datasets so far, this model was only able to be externally validated by independent cohorts with early cervical cancers (IB1 stage). However, the constructed signature was validated by various methods involving subgroup analysis as well as TCGA pan-cancer test as mentioned in this article. This computational frame of Immunoscore identification may also be referred within other cancer types. Therefore, our modeling algorithm was confirmed based on these results and we assumed that this signature might be reliable despite lack of adequate external validation. In the coming future, we will collect clinical specimens to better confirm our conclusion.

To sum up, our study put forward an immune-related signature composed of immune-related genes that were predictive in survival outcome, immune landscape, and response to immunotherapy, regardless of the diversity in detecting methods and platforms.

## Data Availability Statement

The original contributions presented in the study are included in the article/**Supplementary Material**. Further inquiries can be directed to the corresponding authors.

## Author Contributions

JZ and SW conceived and designed the whole project and drafted the manuscript. SY, XL and JZ analyzed the data and wrote the manuscript. SY and XL carried out data interpretations and helped data discussion. JZ and SW revised the manuscript. All authors contributed to the article and approved the submitted version.

## Funding

This study was supported by grants from the National Natural Science Foundation of China (81971340 and 81502230).

## Conflict of Interest

The authors declare that the research was conducted in the absence of any commercial or financial relationships that could be construed as a potential conflict of interest.

## Publisher’s Note

All claims expressed in this article are solely those of the authors and do not necessarily represent those of their affiliated organizations, or those of the publisher, the editors and the reviewers. Any product that may be evaluated in this article, or claim that may be made by its manufacturer, is not guaranteed or endorsed by the publisher.

## Supplementary Material

Supplementary Figure 1 Relationships between expressions of the four immune-related genes in CESC. The Spearman correlation is shown.

Supplementary Figure 2 Kaplan-Meier curves for progression-free survival (PFS) and relapse-free survival (RFS) of CESC patients from TCGA. Log-rank test showed p < 0.05.

Supplementary Figure 3 The association between the Immunoscore and enrichment score of specific gene sets EMT **(A)** and hypoxia **(B)** in TCGA-CESC cohort. Mann-Whitney test, **p<0.01, ***p<0.001

Supplementary Figure 4 The relative mRNA expressions of four immune-related hub genes were tested by quantitative RT-PCR in human cervical epithelial cell line H8 and human cervical cancer cell lines HeLa, SiHa, ME180 and Caski. ****p<0.0001.

Supplementary Figure 5 **(A)** Forest plot of the Immunoscore in all sorts of cancer of TCGA database. **(B, C)** The risk curve and scatter plot of each sample in the TCGA-SKCM and TCGA-HNSC cohorts after realignment *via ggrisk* algorithm. And the heatmap showed distinct expression profiles of four hub genes in the high- and low-risk groups.

Supplementary Figure 6 A univariate Cox hazard ratio analysis revealed that the Immunoscore was statistically different in almost all the subgroups classified by age, gender and tumor stage in SKCM **(A)** and in patients within subgroups such as younger age, male patients as well as III-IV clinical stage in HNSC **(B)**.

Supplementary Figure 7 Spearman correlation analysis of the Immunoscore and tumor-infiltrating cells.

Supplementary Figure 8 Kaplan-Meier curves for overall survival (OS) of all CESC patients with multiple prognostic immune checkpoints. Log-rank test showed p < 0.05 respectively.

Supplementary Figure 9 Expression levels of immunogenic cell death (ICD) modulators in high- and low-score subgroups. The statistical difference of two groups was compared through the Wilcoxon test. *p < 0.05; **p < 0.01; ***p < 0.001; ****p < 0.0001.

Supplementary Figure 10 Gene set enrichment analysis identified hallmark gene sets or immunologic signatures that are activated or suppressed according to their correlation with Immunoscore.

Supplementary Figure 11 Gene set enrichment analysis identified hallmark gene sets or immunologic signatures that are activated or suppressed according to their correlation with the four immune-related genes.

Supplementary Figure 12 **(A)** Scatterplots depicting the negative correlation between GTSF1L expression and clinical biomarkers involving SCC (SERPINB3) and HE4 (WFDC2) in the TCGA-CESC cohort. The Spearman correlation is shown. **(B)** The Pearson correlation analysis of GTSF1L expression and immune checkpoints, such as BTLA and CD47, in all sorts of cancers of TCGA.

Supplementary Figure 13 High amplication of GTSF1L was linked to higher infiltration of CD8^+^ naive T cell and resting mast cell, but correlated with lower infiltration of NK cell and activated mast cell, according to the online tool TIMER 2.0.

Supplementary Figure 14 Spearman correlation analysis of the three immune-related genes (CHIT1, GNG8 and PLA2G2D) and tumor-infiltrating cells.

Supplementary Figure 15 Kaplan-Meier curves for patients in the TCGA-CESC cohort stratified by both GTSF1L expression and levels of immune checkpoints, such as PD1, PD-L1, CTLA-4, CD47 and CD38 and BTLA. Log-rank test, p<0.01.
