# Supplementary figures and images for: Development of a Novel Immune Infiltration-Based Gene Signature to Predict Prognosis and Immunotherapy Response of Patients With Cervical Cancer
Source: Front Immunol. 2021 Sep 3;12:709493. doi: 10.3389/fimmu.2021.709493 (PMC8446628; doi:10.3389/fimmu.2021.709493)

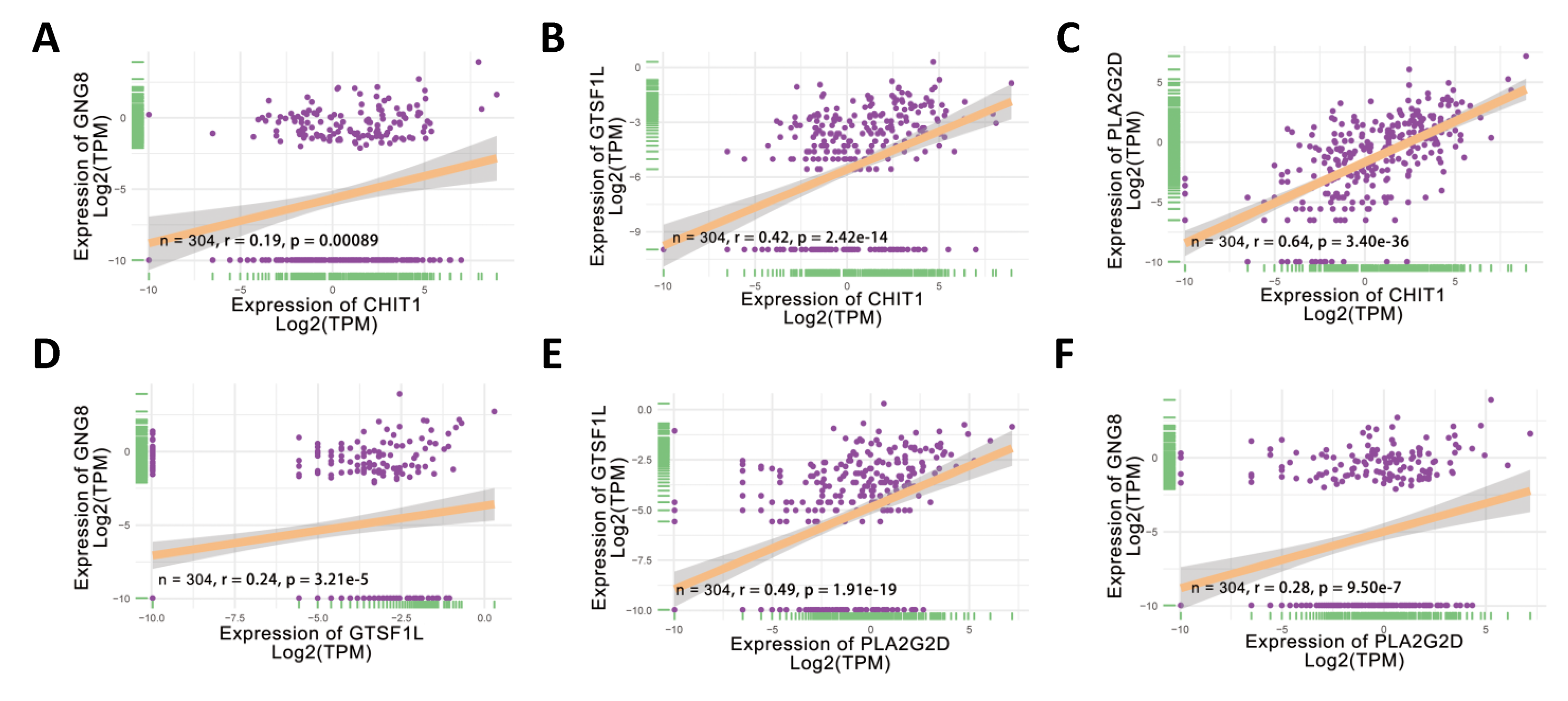

Supplement: Supplementary Figure 1 — Relationships between expressions of the four immune-related genes in CESC. The Spearman correlation is shown. [file Image_1.tif]

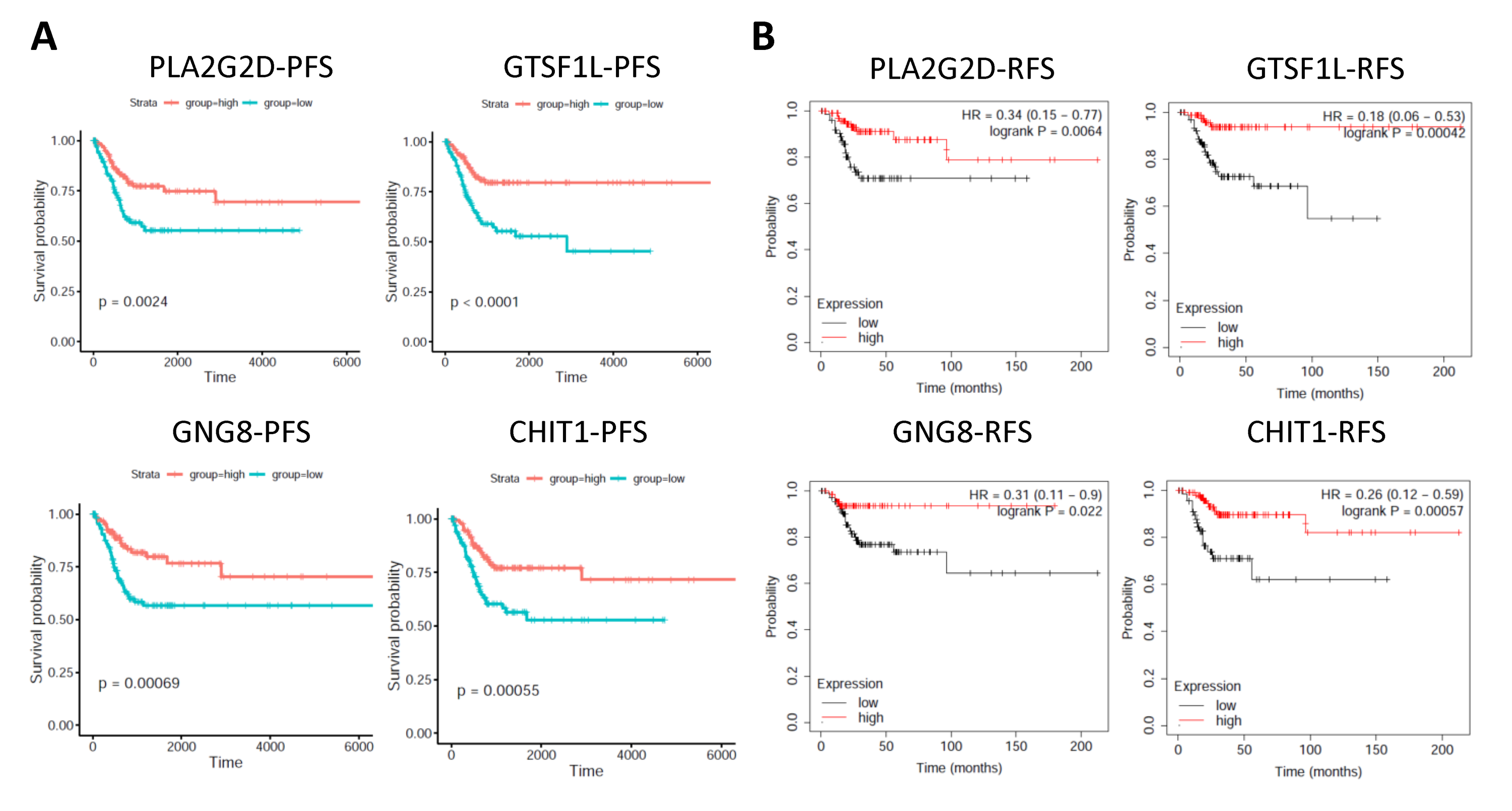

Supplement: Supplementary Figure 2 — Kaplan-Meier curves for progression-free survival (PFS) and relapse-free survival (RFS) of CESC patients from TCGA. Log-rank test showed p < 0.05. [file Image_2.tif]

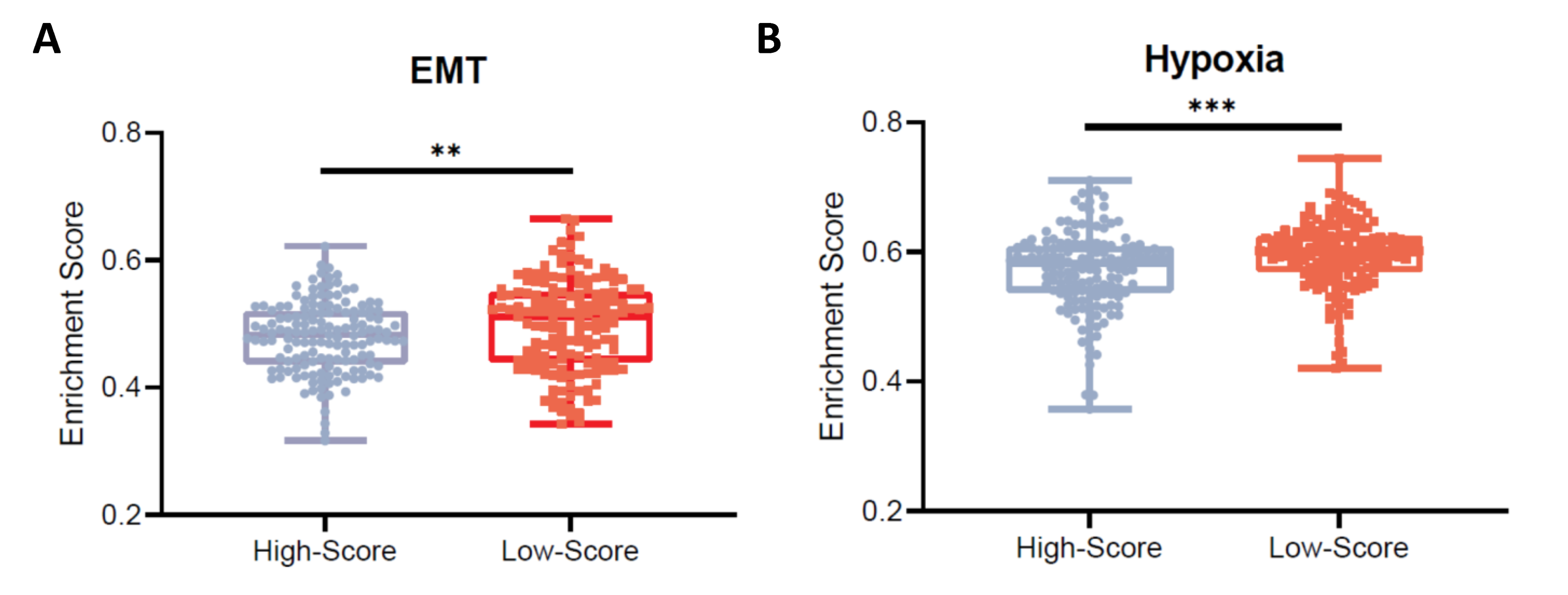

Supplement: Supplementary Figure 3 — The association between the Immunoscore and enrichment score of specific gene sets EMT (A) and hypoxia (B) in TCGA-CESC cohort. Mann-Whitney test, **p<0.01, ***p<0.001 [file Image_3.tif]

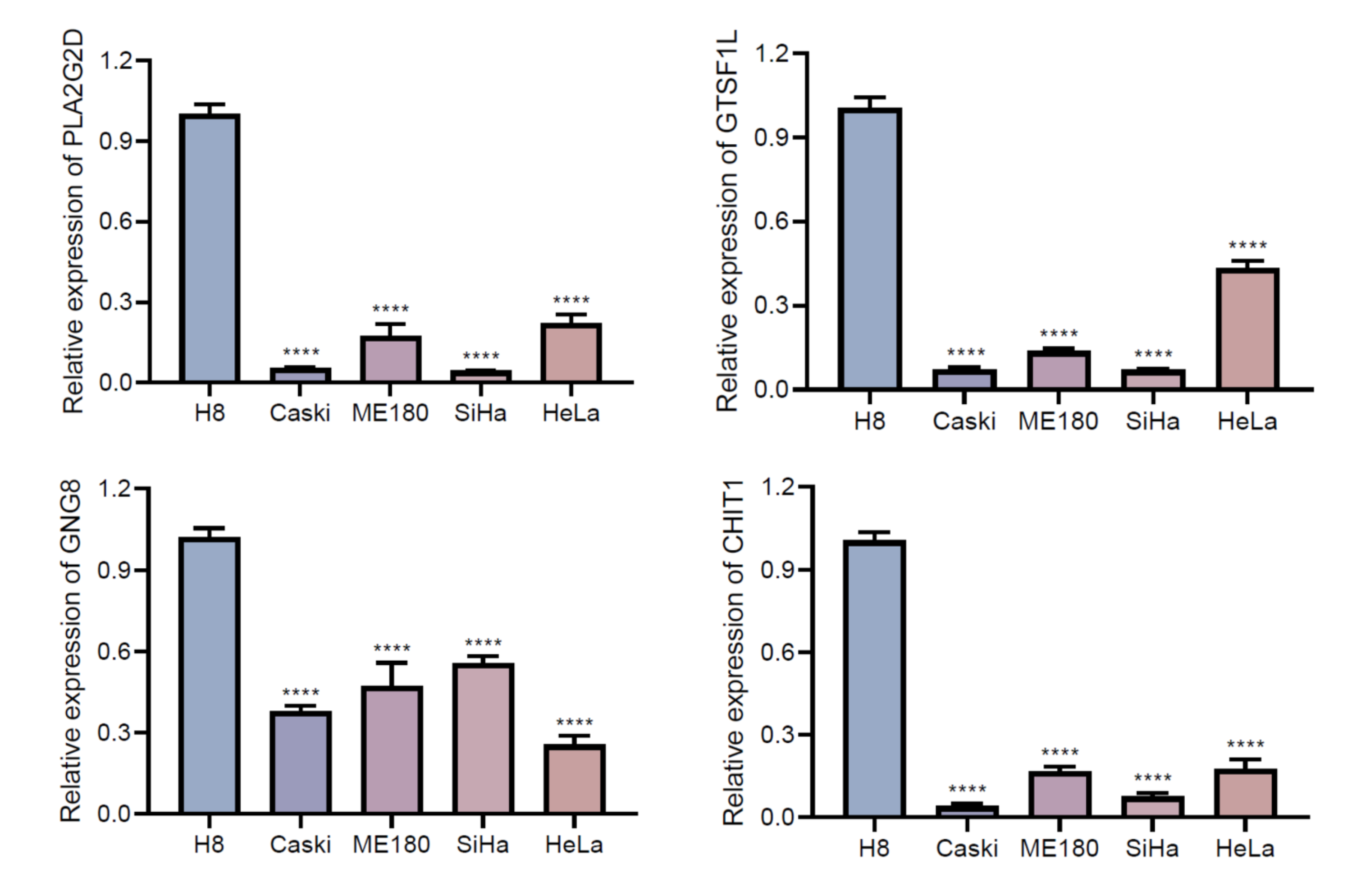

Supplement: Supplementary Figure 4 — The relative mRNA expressions of four immune-related hub genes were tested by quantitative RT-PCR in human cervical epithelial cell line H8 and human cervical cancer cell lines HeLa, SiHa, ME180 and Caski. ****p<0.0001. [file Image_4.tif]

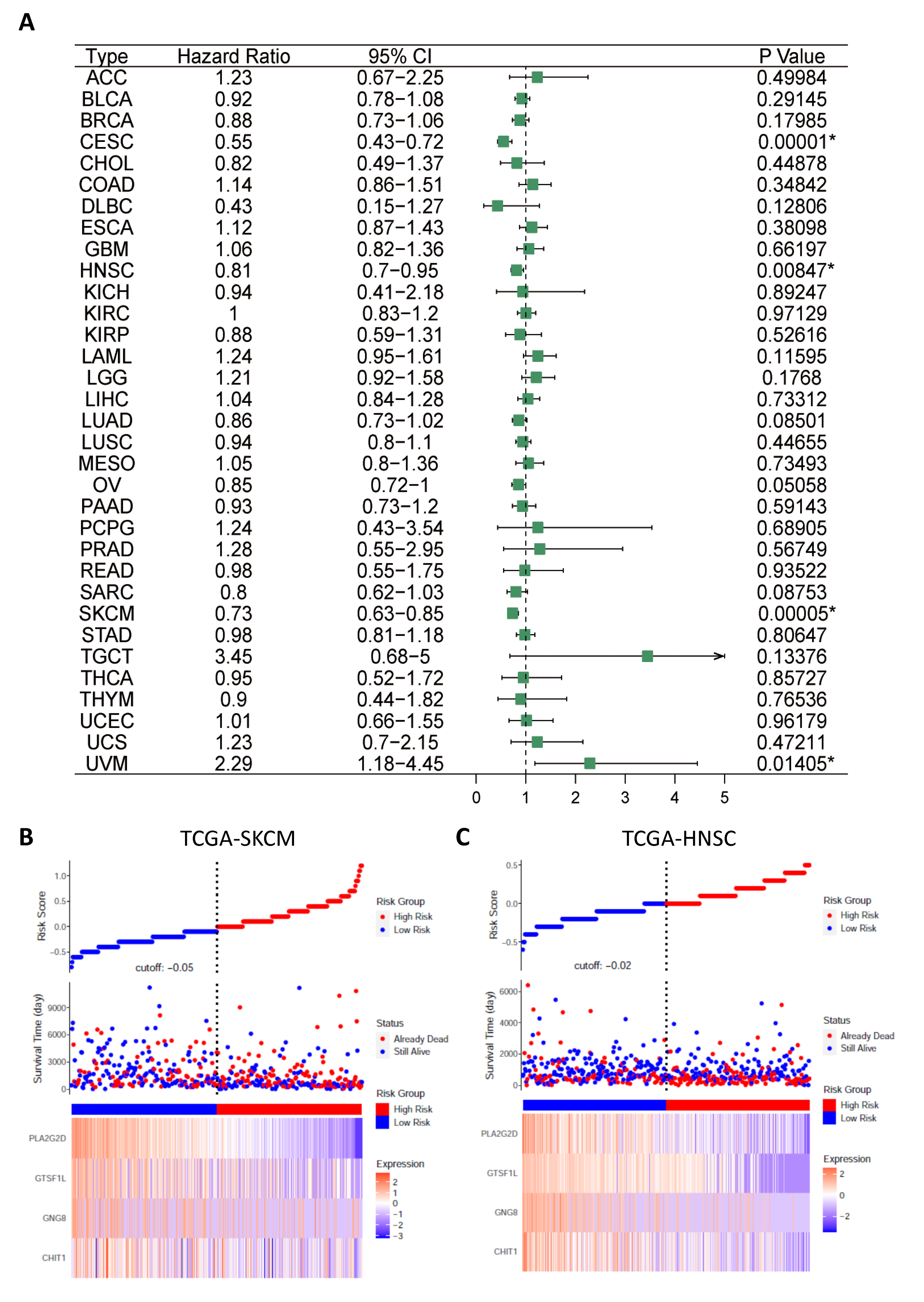

Supplement: Supplementary Figure 5 — (A) Forest plot of the Immunoscore in all sorts of cancer of TCGA database. (B, C) The risk curve and scatter plot of each sample in the TCGA-SKCM and TCGA-HNSC cohorts after realignment via ggrisk algorithm. And the heatmap showed distinct expression profiles of four hub genes in the high- and low-risk groups. [file Image_5.tif]

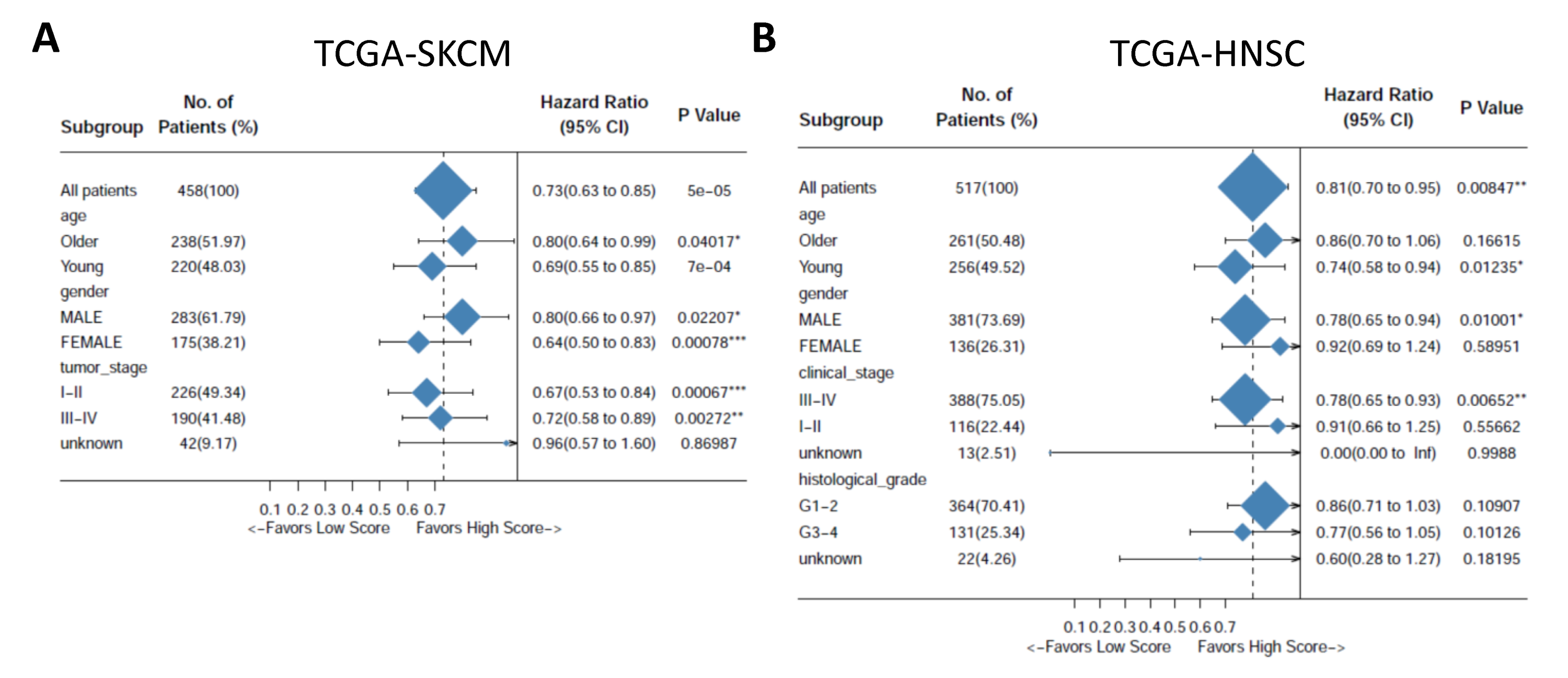

Supplement: Supplementary Figure 6 — A univariate Cox hazard ratio analysis revealed that the Immunoscore was statistically different in almost all the subgroups classified by age, gender and tumor stage in SKCM (A) and in patients within subgroups such as younger age, male patients as well as III-IV clinical stage in HNSC (B). [file Image_6.tif]

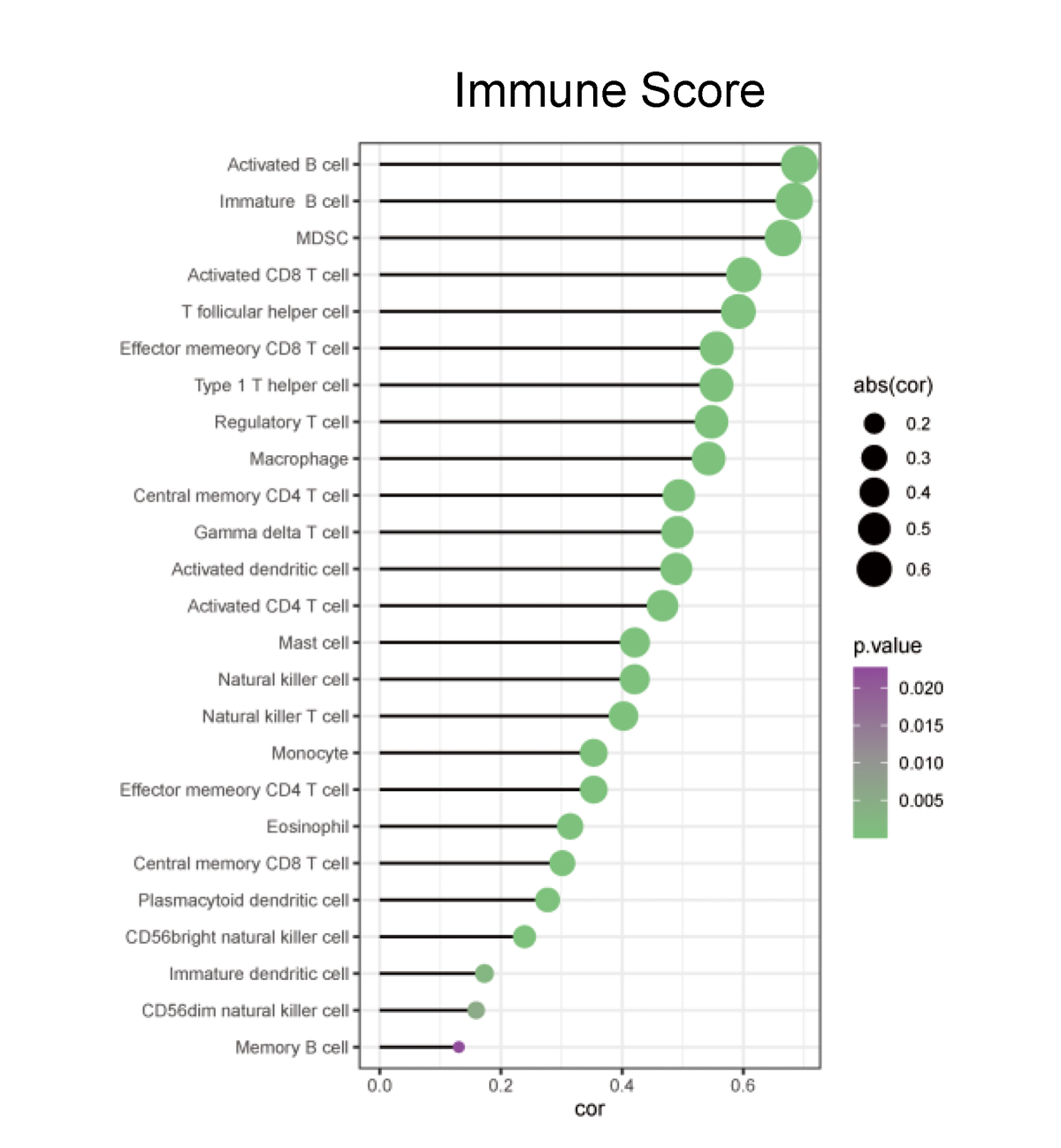

Supplement: Supplementary Figure 7 — Spearman correlation analysis of the Immunoscore and tumor-infiltrating cells. [file Image_7.tif]

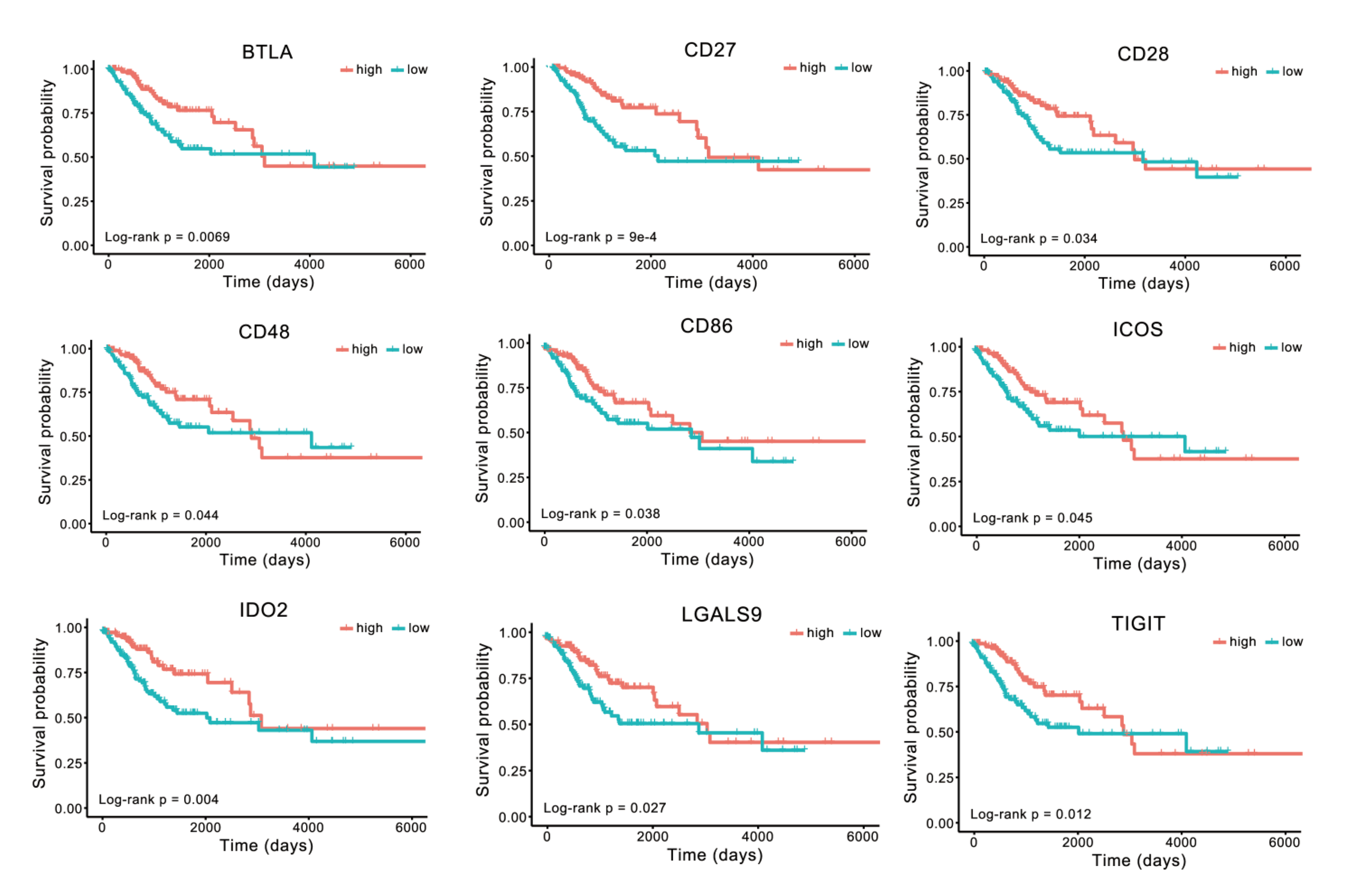

Supplement: Supplementary Figure 8 — Kaplan-Meier curves for overall survival (OS) of all CESC patients with multiple prognostic immune checkpoints. Log-rank test showed p < 0.05 respectively. [file Image_8.tif]

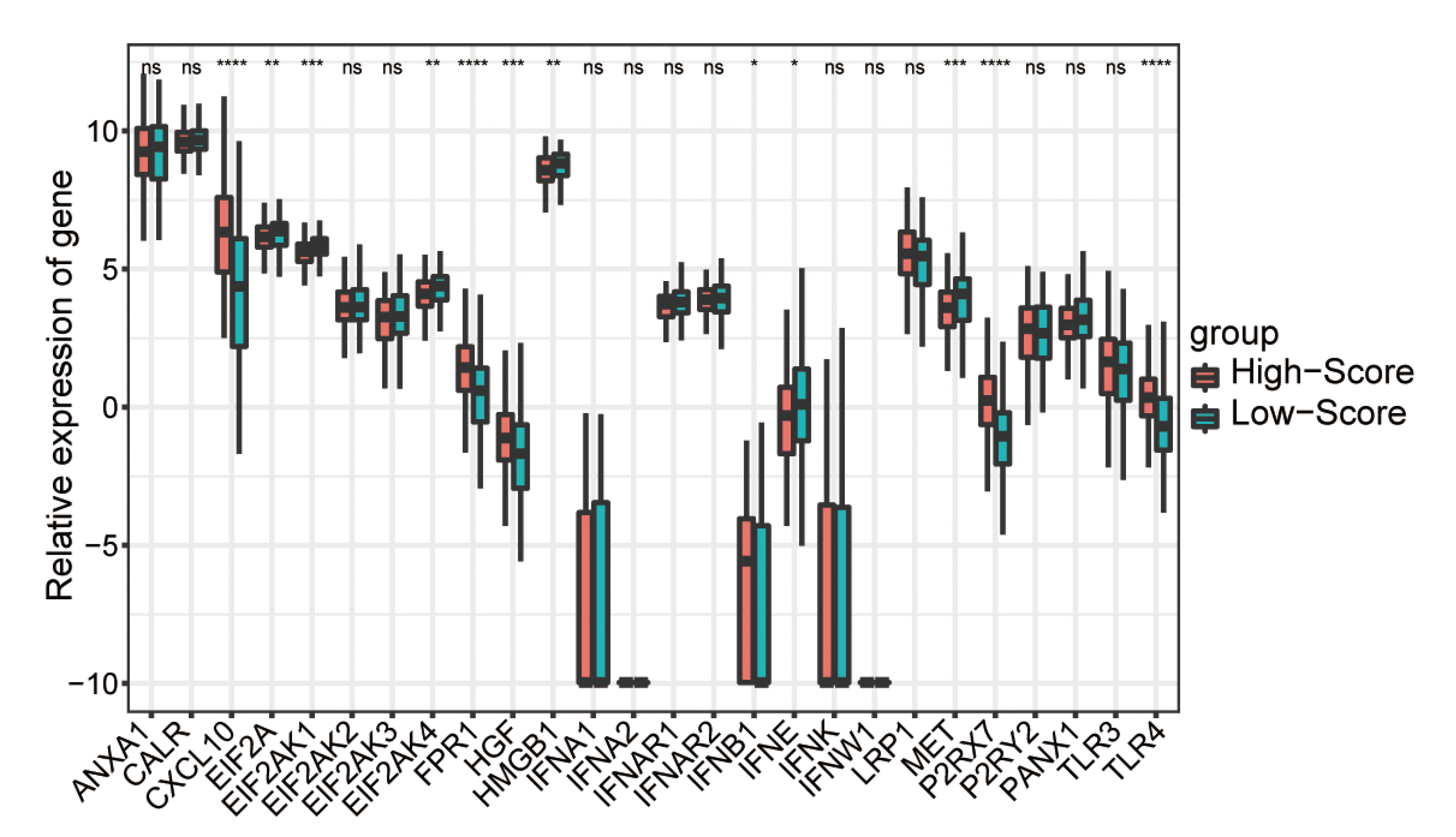

Supplement: Supplementary Figure 9 — Expression levels of immunogenic cell death (ICD) modulators in high- and low-score subgroups. The statistical difference of two groups was compared through the Wilcoxon test. *p < 0.05; **p < 0.01; ***p < 0.001; ****p < 0.0001. [file Image_9.tif]

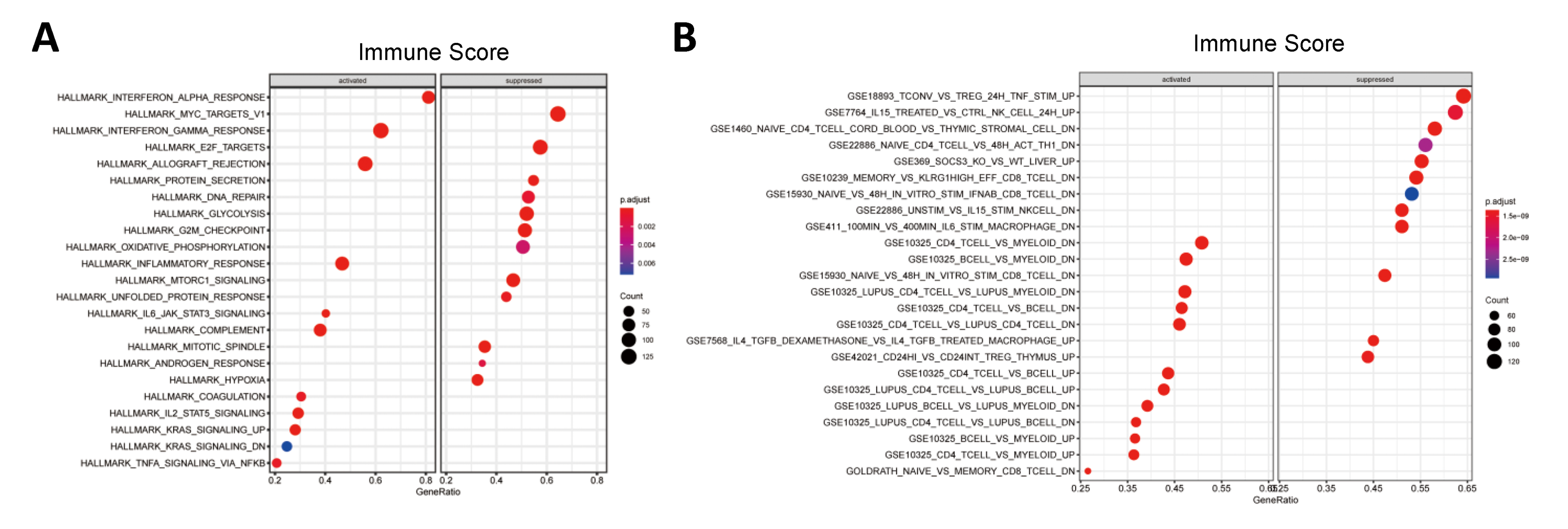

Supplement: Supplementary Figure 10 — Gene set enrichment analysis identified hallmark gene sets or immunologic signatures that are activated or suppressed according to their correlation with Immunoscore. [file Image_10.tif]

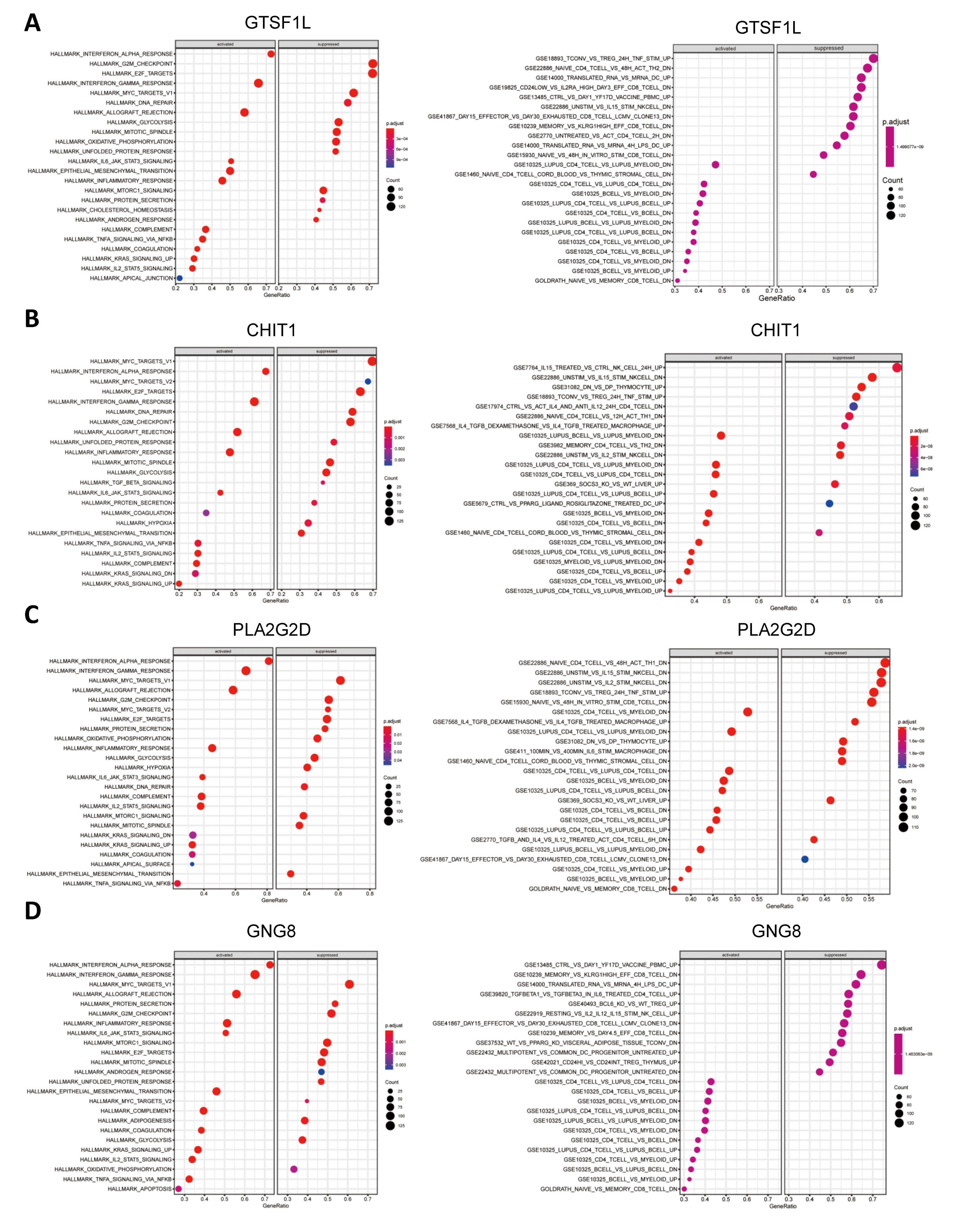

Supplement: Supplementary Figure 11 — Gene set enrichment analysis identified hallmark gene sets or immunologic signatures that are activated or suppressed according to their correlation with the four immune-related genes. [file Image_11.tif]

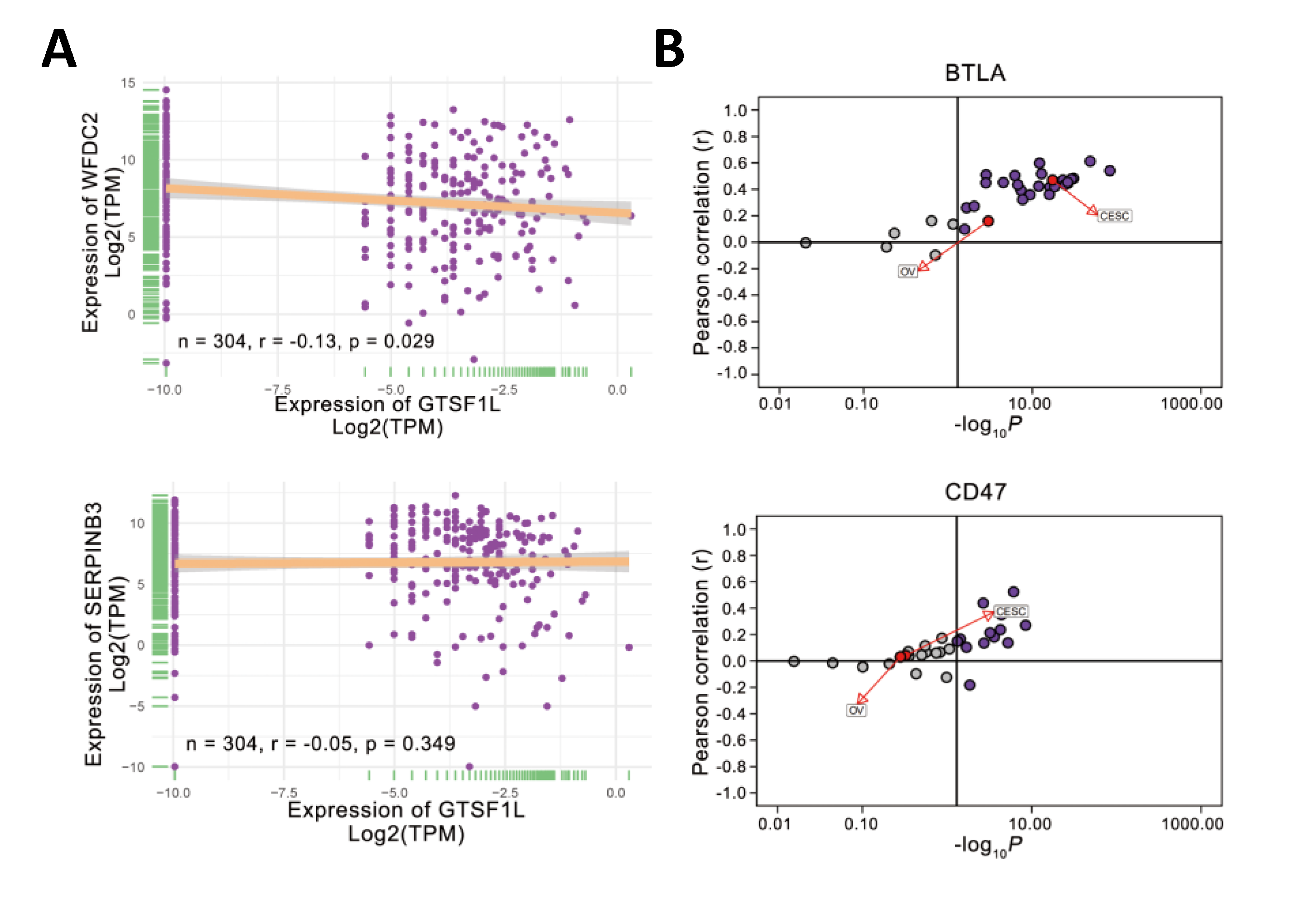

Supplement: Supplementary Figure 12 — (A) Scatterplots depicting the negative correlation between GTSF1L expression and clinical biomarkers involving SCC (SERPINB3) and HE4 (WFDC2) in the TCGA-CESC cohort. The Spearman correlation is shown. (B) The Pearson correlation analysis of GTSF1L expression and immune checkpoints, such as BTLA and CD47, in all sorts of cancers of TCGA. [file Image_12.tif]

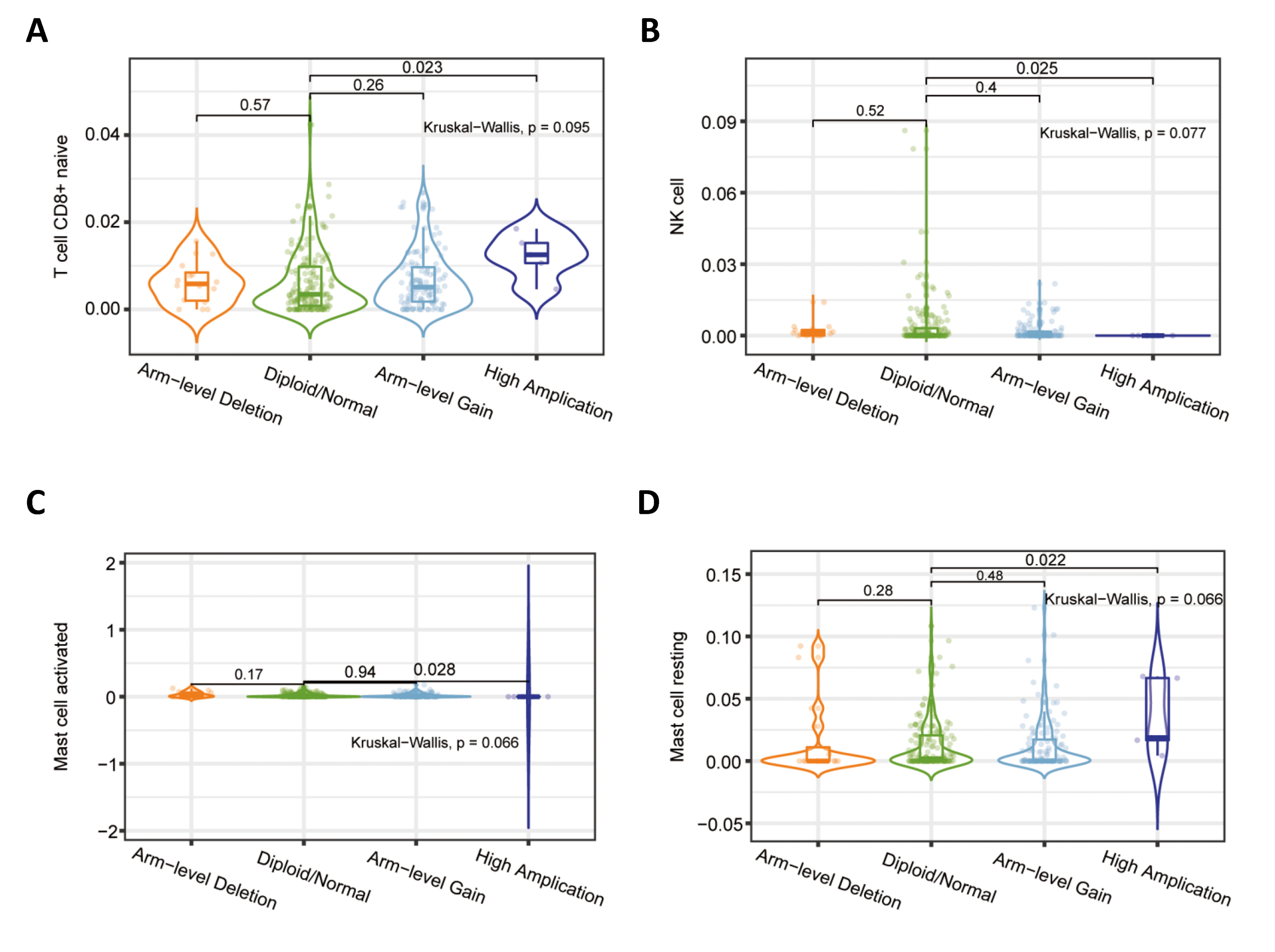

Supplement: Supplementary Figure 13 — High amplication of GTSF1L was linked to higher infiltration of CD8+ naive T cell and resting mast cell, but correlated with lower infiltration of NK cell and activated mast cell, according to the online tool TIMER 2.0. [file Image_13.tif]

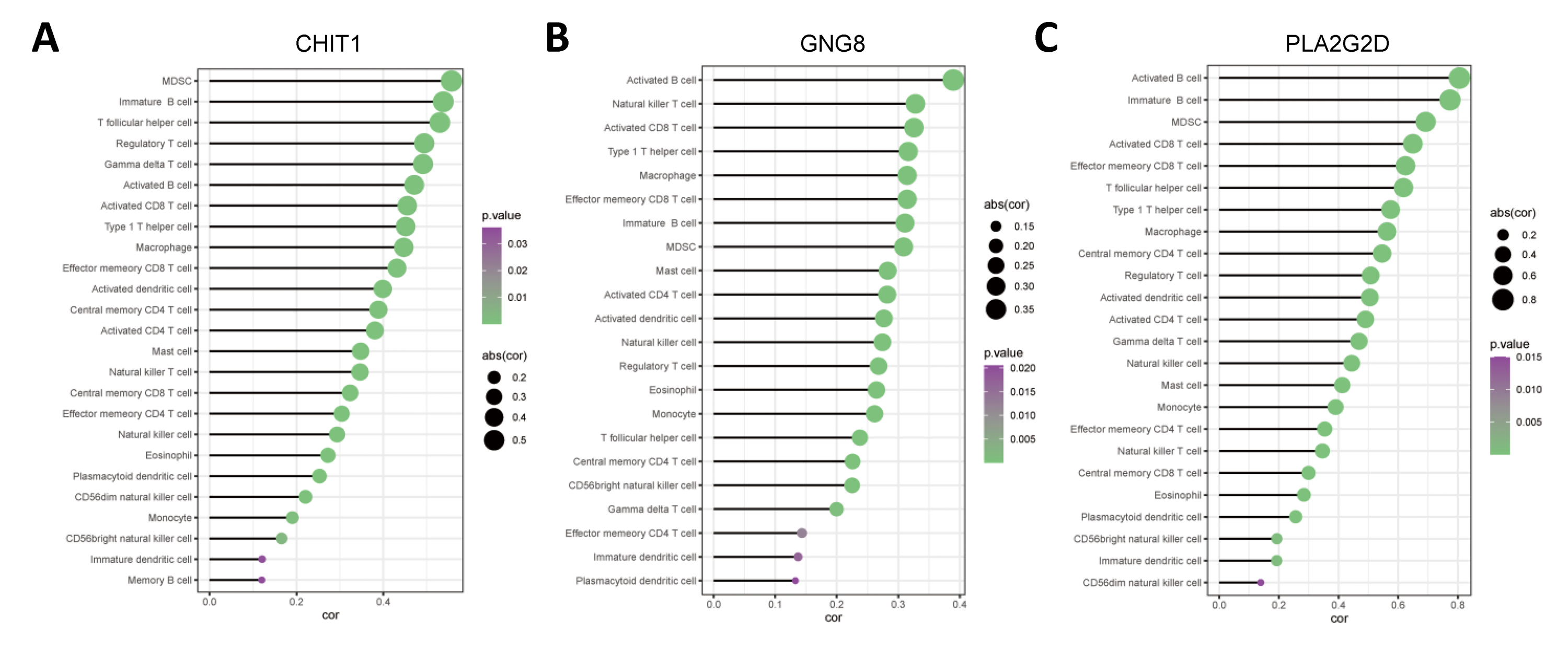

Supplement: Supplementary Figure 14 — Spearman correlation analysis of the three immune-related genes (CHIT1, GNG8 and PLA2G2D) and tumor-infiltrating cells. [file Image_14.tif]

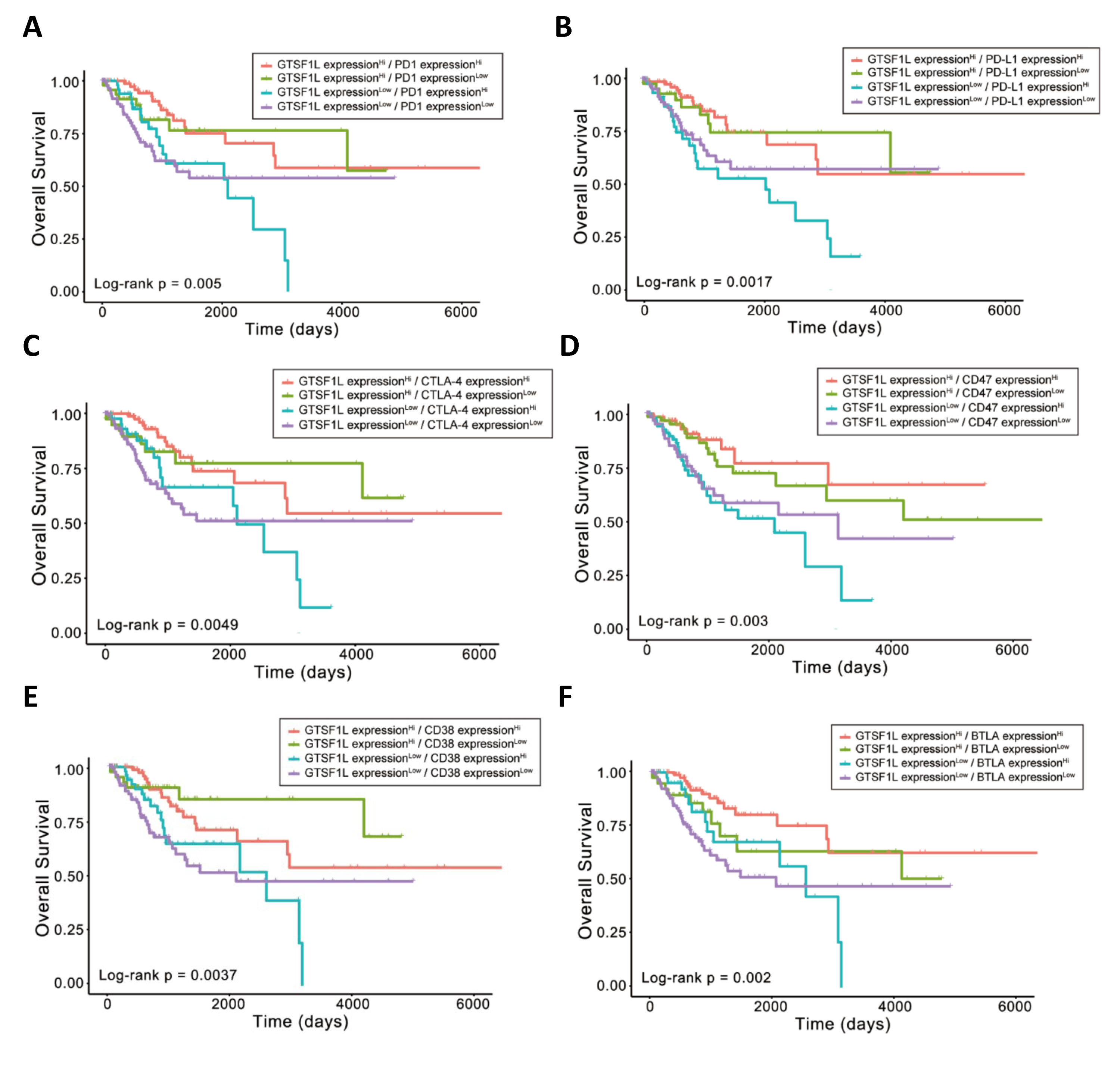

Supplement: Supplementary Figure 15 — Kaplan-Meier curves for patients in the TCGA-CESC cohort stratified by both GTSF1L expression and levels of immune checkpoints, such as PD1, PD-L1, CTLA-4, CD47 and CD38 and BTLA. Log-rank test, p<0.01. [file Image_15.tif]
