# Supplementary material for: Development of a Novel Immune Infiltration-Based Gene Signature to Predict Prognosis and Immunotherapy Response of Patients With Cervical Cancer
Source: Front Immunol. 2021 Sep 3;12:709493. doi: 10.3389/fimmu.2021.709493 (PMC8446628; doi:10.3389/fimmu.2021.709493)
Supplement: Supplementary file 16 [file Table_1.docx]

**Supplementary Table S1. Sequences of primers for qRT-PCR.**

| Gene Symbol | Sequences 5’-3’ |
| --- | --- |
| CHIT1 | Forward: CTTCTTCTGAGTGCAGCGGTTC  Reverse: AGGTTCTGGGCGATTTTGTC |
| GTSF1L | Forward: CATCTGCTATAGTTCTGTCTCGG  Reverse: CTTGGGATTCTGTAGGTGACG |
| PLA2G2D | Forward: CAAAGCAACACCAGAACCAAG  Reverse: CCCCTTGTAATTCACCCTGAG |
| GNG8 | Forward: TGAAGCTGGAGGTGAACATC  Reverse: TCATCTTTGGCATGCGTCTC |
| GAPDH | Forward: CAACAGCGACACCCACTC  Reverse: ATCACGCCACAGTTTCCC |
