# Supplementary material for: Development of a Novel Immune Infiltration-Based Gene Signature to Predict Prognosis and Immunotherapy Response of Patients With Cervical Cancer
Source: Front Immunol. 2021 Sep 3;12:709493. doi: 10.3389/fimmu.2021.709493 (PMC8446628; doi:10.3389/fimmu.2021.709493)
Supplement: Supplementary file 17 [file Table_2.docx]

**Supplementary Table S2. Univariate and multi-variate Cox regression proportional hazards model to analyze the correlation between the expression status of 60 genes and overall survival.**

|  | Univariate | | | Multivariate | | |
| --- | --- | --- | --- | --- | --- | --- |
| Gene | Hazard Ratio | CI95 | P value | Hazard Ratio | CI95 | P value |
| PLA2G2D | 0.47 | 0.29-0.75 | 0.00171 | 0.18 | 0.06-0.52 | 0.00146 |
| CHIT1 | 0.51 | 0.32-0.82 | 0.00551 | 0.34 | 0.14-0.82 | 0.0159 |
| GTSF1L | 0.42 | 0.25-0.69 | 0.00056 | 0.43 | 0.22-0.87 | 0.01794 |
| GNG8 | 0.51 | 0.31-0.83 | 0.00667 | 0.53 | 0.29-0.98 | 0.0427 |
| CCL19 | 0.74 | 0.46-1.19 | 0.21202 | 6.17 | 2.03-18.73 | 0.00131 |
| KCNA3 | 0.72 | 0.45-1.15 | 0.1655 | 3 | 1.08-8.3 | 0.03484 |
| IGLL5 | 0.7 | 0.44-1.12 | 0.13587 | 3 | 0.92-9.79 | 0.06818 |
| CCR7 | 0.45 | 0.28-0.73 | 0.00124 | 0.46 | 0.2-1.07 | 0.07009 |
| GH1 | 0.6 | 0.36-0.99 | 0.04456 | 0.54 | 0.28-1.07 | 0.07591 |
| LY9 | 0.47 | 0.29-0.77 | 0.00235 | 0.41 | 0.16-1.1 | 0.07721 |
| P2RY12 | 0.72 | 0.45-1.15 | 0.16366 | 2.04 | 0.92-4.52 | 0.07753 |
| MYBPC2 | 0.5 | 0.3-0.83 | 0.00765 | 0.58 | 0.3-1.12 | 0.10636 |
| TIFAB | 0.69 | 0.43-1.11 | 0.12703 | 2.28 | 0.81-6.38 | 0.11744 |
| HMHB1 | 0.62 | 0.33-1.15 | 0.13107 | 0.54 | 0.25-1.17 | 0.11801 |
| PTCRA | 0.76 | 0.48-1.21 | 0.24487 | 1.86 | 0.83-4.14 | 0.12993 |
| BFSP2 | 0.73 | 0.46-1.17 | 0.19115 | 1.66 | 0.81-3.37 | 0.16313 |
| CLEC6A | 0.7 | 0.44-1.12 | 0.14199 | 1.76 | 0.78-3.99 | 0.17504 |
| JCHAIN | 0.57 | 0.35-0.92 | 0.02105 | 0.43 | 0.13-1.47 | 0.1782 |
| CD1C | 0.49 | 0.31-0.8 | 0.004 | 0.55 | 0.23-1.32 | 0.18357 |
| CXorf65 | 0.56 | 0.35-0.9 | 0.01716 | 0.65 | 0.33-1.31 | 0.23219 |
| CCR6 | 0.73 | 0.45-1.17 | 0.1852 | 1.51 | 0.68-3.32 | 0.3085 |
| AMPD1 | 0.6 | 0.37-0.97 | 0.03906 | 0.63 | 0.25-1.58 | 0.32789 |
| FCER2 | 0.64 | 0.39-1.02 | 0.06186 | 1.47 | 0.65-3.29 | 0.35248 |
| BLK | 0.71 | 0.45-1.14 | 0.15646 | 1.49 | 0.58-3.86 | 0.40789 |
| LCN6 | 0.59 | 0.37-0.95 | 0.03128 | 0.73 | 0.34-1.56 | 0.42118 |
| TCL1A | 0.57 | 0.36-0.92 | 0.02187 | 0.73 | 0.3-1.78 | 0.48587 |
| CRLF2 | 0.71 | 0.44-1.15 | 0.16127 | 0.82 | 0.45-1.5 | 0.52578 |
| CD1E | 0.5 | 0.31-0.81 | 0.005 | 0.73 | 0.26-2.03 | 0.54822 |
| MZB1 | 0.7 | 0.44-1.12 | 0.13329 | 0.73 | 0.23-2.33 | 0.59853 |
| CLEC10A | 0.64 | 0.4-1.02 | 0.05818 | 1.26 | 0.51-3.11 | 0.61559 |
| CD1A | 0.57 | 0.35-0.91 | 0.01946 | 0.79 | 0.32-1.98 | 0.61697 |
| ACKR1 | 0.62 | 0.39-1 | 0.04771 | 0.84 | 0.36-1.96 | 0.68205 |
| ANKRD55 | 0.54 | 0.33-0.89 | 0.01537 | 1.17 | 0.54-2.51 | 0.69296 |
| FCRL5 | 0.74 | 0.46-1.18 | 0.20608 | 0.82 | 0.27-2.49 | 0.72555 |
| CCL25 | 0.65 | 0.41-1.05 | 0.07856 | 0.89 | 0.45-1.76 | 0.74107 |
| CD79A | 0.6 | 0.37-0.96 | 0.03375 | 0.82 | 0.23-2.92 | 0.76084 |
| CD1B | 0.58 | 0.36-0.93 | 0.02423 | 1.13 | 0.47-2.7 | 0.78177 |
| LGALS2 | 0.68 | 0.43-1.09 | 0.11219 | 1.11 | 0.51-2.41 | 0.79109 |
| RGS13 | 0.67 | 0.41-1.08 | 0.10238 | 0.92 | 0.48-1.77 | 0.79732 |
| PSMA8 | 0.66 | 0.41-1.07 | 0.09034 | 0.92 | 0.47-1.79 | 0.7979 |
| TNFRSF13B | 0.72 | 0.45-1.15 | 0.17332 | 1.1 | 0.44-2.75 | 0.84627 |
| ZNF831 | 0.49 | 0.3-0.79 | 0.00368 | 0.91 | 0.31-2.67 | 0.86879 |
| PNOC | 0.66 | 0.41-1.06 | 0.08801 | 0.94 | 0.31-2.89 | 0.9203 |
| CADM3 | 0.68 | 0.43-1.1 | 0.11551 | 0.97 | 0.49-1.93 | 0.93672 |
| CLEC4C | 0.61 | 0.38-0.98 | 0.04073 | 1.02 | 0.47-2.24 | 0.9557 |
| GZMK | 0.58 | 0.36-0.94 | 0.02663 | 1.01 | 0.43-2.35 | 0.98804 |
| ZNF80 | 0.63 | 0.39-1.02 | 0.05788 | 1 | 0.5-2 | 0.98967 |
| CCL21 | 0.87 | 0.55-1.39 | 0.57111 | / | / | / |
| CCL23 | 0.82 | 0.51-1.31 | 0.4085 | / | / | / |
| FCRL2 | 0.79 | 0.5-1.27 | 0.33277 | / | / | / |
| FCRLA | 1 | 0.63-1.6 | 0.99175 | / | / | / |
| FLT3 | 0.82 | 0.52-1.31 | 0.41469 | / | / | / |
| IL2 | 0.84 | 0.53-1.34 | 0.46712 | / | / | / |
| MAL | 0.8 | 0.5-1.28 | 0.35966 | / | / | / |
| MEIKIN | 0.79 | 0.49-1.26 | 0.31577 | / | / | / |
| MS4A1 | 0.78 | 0.49-1.24 | 0.2884 | / | / | / |
| NME8 | 0.83 | 0.52-1.32 | 0.42674 | / | / | / |
| P2RY10 | 0.83 | 0.52-1.32 | 0.41932 | / | / | / |
| PVALB | 1.26 | 0.79-2.01 | 0.33437 | / | / | / |
| TNFRSF17 | 0.77 | 0.48-1.23 | 0.2755 | / | / | / |
