# Supplementary material for: Development of a Novel Immune Infiltration-Based Gene Signature to Predict Prognosis and Immunotherapy Response of Patients With Cervical Cancer
Source: Front Immunol. 2021 Sep 3;12:709493. doi: 10.3389/fimmu.2021.709493 (PMC8446628; doi:10.3389/fimmu.2021.709493)
Supplement: Supplementary file 18 [file Table_3.docx]

**Supplementary Table S3. Results of univariate and multi-variate Cox regression analyses between the Immunoscore signature as well as patient age and overall survival.**

|  | Univariate | | | Multivariate | | |
| --- | --- | --- | --- | --- | --- | --- |
| Characteristics | Hazard Ratio | CI95 | P value | Hazard Ratio | CI95 | P value |
| Age | 1.02 | 1-1.03 | 0.05702 | 1.02 | 1-1.04 | 0.02591 |
| Immunoscore | 0.55 | 0.43-0.72 | 0.00001 | 0.53 | 0.41-0.7 | 0.00001 |
